# Aberrant methylation of tRNAs links cellular stress to neuro-developmental disorders

**DOI:** 10.15252/embj.201489282

**Published:** 2014-07-25

**Authors:** Sandra Blanco, Sabine Dietmann, Joana V Flores, Shobbir Hussain, Claudia Kutter, Peter Humphreys, Margus Lukk, Patrick Lombard, Lucas Treps, Martyna Popis, Stefanie Kellner, Sabine M Hölter, Lillian Garrett, Wolfgang Wurst, Lore Becker, Thomas Klopstock, Helmut Fuchs, Valerie Gailus-Durner, Martin Hrabĕ de Angelis, Ragnhildur T Káradóttir, Mark Helm, Jernej Ule, Joseph G Gleeson, Duncan T Odom, Michaela Frye

**Affiliations:** 1 Wellcome Trust – Medical Research Council Cambridge Stem Cell Institute, University of CambridgeCambridge, UK; 2 Li Ka Shing Centre, CR-UK Cambridge Institute, University of CambridgeCambridge, UK; 3 CNRS, UMR8104Paris, France; 4 Johannes Gutenberg University Mainz, Institute for Pharmacy and BiochemistryMainz, Germany; 5 German Mouse Clinic, Helmholtz Zentrum MünchenNeuherberg, Germany; 6 Institute of Developmental Genetics, Helmholtz Zentrum MünchenNeuherberg, Germany; 7 German Center for Vertigo and Balance DisordersMunich, Germany; 8 Institute for Experimental Genetics, Helmholtz Zentrum MünchenNeuherberg, Germany; 9 Department of Neurology, Friedrich-Baur-Institute, Ludwig-Maximilians-UniversityMunich, Germany; 10 Department of Molecular Neuroscience, UCL Institute of NeurologyLondon, UK; 11 Laboratory of Pediatric Brain Diseases, Howard Hughes Medical Institute, The Rockefeller UniversityNew York, NY, USA

**Keywords:** 5-methylcytidine, Misu, NSun2, RNA modification

## Abstract

Mutations in the cytosine-5 RNA methyltransferase NSun2 cause microcephaly and other neurological abnormalities in mice and human. How post-transcriptional methylation contributes to the human disease is currently unknown. By comparing gene expression data with global cytosine-5 RNA methylomes in patient fibroblasts and NSun2-deficient mice, we find that loss of cytosine-5 RNA methylation increases the angiogenin-mediated endonucleolytic cleavage of transfer RNAs (tRNA) leading to an accumulation of 5′ tRNA-derived small RNA fragments. Accumulation of 5′ tRNA fragments in the absence of NSun2 reduces protein translation rates and activates stress pathways leading to reduced cell size and increased apoptosis of cortical, hippocampal and striatal neurons. Mechanistically, we demonstrate that angiogenin binds with higher affinity to tRNAs lacking site-specific NSun2-mediated methylation and that the presence of 5′ tRNA fragments is sufficient and required to trigger cellular stress responses. Furthermore, the enhanced sensitivity of NSun2-deficient brains to oxidative stress can be rescued through inhibition of angiogenin during embryogenesis. In conclusion, failure in NSun2-mediated tRNA methylation contributes to human diseases via stress-induced RNA cleavage.

See also: **G Stoecklin & S Diederichs** (September 2014)

## Introduction

Hereditary forms of intellectual disability (ID) are neuro-developmental disorders with a worldwide prevalence of around 2% (Ropers, 2008; de Ligt *et al*, 2012). Several genetic mutations in the NSUN2 gene have been identified to cause a syndromic form of intellectual disability and a Dubowitz-like syndrome in humans (Abbasi-Moheb *et al*, 2012; Khan *et al*, 2012; Martinez *et al*, 2012). In individuals with the Dubowitz-like syndrome, the NSUN2 gene carries a homozygous mutation in the canonical splice acceptor site of exon 6 leading to a truncation of the transcribed mRNA and loss of the NSun2 protein (Martinez *et al*, 2012). Besides ID, the patients show further symptoms of neurological abnormalities such as microcephaly, behavioural deficits, speech delay, abnormal gait as well as morphological features including growth retardation, unusual facies and cutaneous abnormalities (Dubowitz, 1965; Huber *et al*, 2011; Martinez *et al*, 2012). While some of the morphological symptoms are also present in loss-of-function transgenic mouse models for NSun2, neurological defects have not been studied yet (Blanco *et al*, 2011). However, loss of the NSun2 orthologue in *Drosophila* causes severe short-term memory deficits (Abbasi-Moheb *et al*, 2012). It is known that NSun2 is a conserved RNA methyltransferase that modifies cytosine-5 in transfer RNAs (tRNAs) and other non-coding RNA species (Brzezicha *et al*, 2006; Frye & Watt, 2006; Blanco *et al*, 2011; Hussain *et al*, 2013b; Khoddami & Cairns, 2013), but whether and how loss of RNA methylation can cause symptoms of these complex diseases is currently unknown.

Although cytosine-5 methylation (m^5^C) is one of the best characterised epigenetic modifications found in DNA (Suzuki & Bird, 2008), the cellular and molecular functions of the same modified nucleobase in RNA remain unclear. Deletion of cytosine-5 tRNA methyltransferases in yeast, flies, fish and mice is not lethal (Wu *et al*, 1998; Goll *et al*, 2006; Rai *et al*, 2007; Chernyakov *et al*, 2008; Blanco *et al*, 2011). However, loss of certain tRNA modifications can increase sensitivity to stress stimuli, including drugs, DNA damage or environmental cues (Wu *et al*, 1998; Jablonowski *et al*, 2006; Begley *et al*, 2007; Schaefer *et al*, 2010). The three currently known cytosine-5 tRNA methyltransferases in higher eukaryotes are NSun2, NSun4 and Dnmt2 (Brzezicha *et al*, 2006; Frye & Watt, 2006; Schaefer *et al*, 2010; Blanco *et al*, 2011; Tuorto *et al*, 2012; Metodiev *et al*, 2014). While NSun4 ablation is lethal in mice, Dnmt2-deficient mice do not exhibit any gross phenotype (Goll *et al*, 2006; Metodiev *et al*, 2014). Deletion of NSun2 alone or in combination with Dnmt2 can impair cellular differentiation pathways in skin, testes and brain (Rai *et al*, 2007; Blanco *et al*, 2011; Tuorto *et al*, 2012; Hussain *et al*, 2013c).

Lack of Dnmt2-mediated methylation in flies increases stress-induced cleavage of tRNAs and sensitises flies to oxidative stress *in vivo* (Schaefer *et al*, 2010). In humans, stress-induced tRNA cleavage is mediated by angiogenin and although Dnmt2 methylation protects tRNAs from angiogenin cleavage *in vitro*, its biological function in humans has yet to be identified (Yamasaki *et al*, 2009; Schaefer *et al*, 2010). Together, these data provide compelling evidence that site-specific methylation of tRNAs protects from angiogenin-mediated cleavage. However, it is currently unknown how non-Dnmt2-targeted tRNAs are protected from cleavage. Dnmt2-mediated methylation is specific to the three tRNAs Gly^GCC^, Asp^GTC^ and Val^AAC^ (Schaefer *et al*, 2010; Tuorto *et al*, 2012; Khoddami & Cairns, 2013), yet all tRNA isoacceptors can be cleaved.

So far, no human disease has been associated with mutations in Dnmt2, but similar to NSun2, also mutations in angiogenin are linked to neurological disorders (van Es *et al*, 2011). Neuro-developmental and intellectual disabilities are further commonly associated with oxidative stress (Wu *et al*, 2007; De Felice *et al*, 2012; Lintas *et al*, 2012).

Here, we set out to answer the fundamental question how hypo-methylated RNA can cause human neurological disorders. By combining genome-wide methylation approaches with *in vitro* and *in vivo* assays in NSun2 knockout mouse models and cells obtained from individuals with Dubowitz-like syndrome, we show that cytosine-5 tRNA methylation is a very common modification and is required to mediate cellular survival during stress responses. We reveal that fragmentation of tRNAs into short non-coding RNAs is perturbed in mouse and patient cells lacking the NSun2 protein, and identify aberrant accumulation of cleaved tRNAs as one mechanism by which mutations in a RNA methyltransferase can result in neurological abnormalities in mice and humans.

## Results

### Cytosine-5 methylation is a common modification in actively transcribed tRNAs

Recent high-throughput RNA methylation profiling identified tRNAs as the most common cytosine-5 methylated RNA species (Squires *et al*, 2012; Hussain *et al*, 2013b; Khoddami & Cairns, 2013). However, the identification of the specific methylated tRNAs has proven difficult because there are over 400 predicted tRNA genes in the mammalian genome, which are highly similar and repetitive in DNA sequence (Lowe & Eddy, 1997). Our first aim was therefore to precisely map cytosine-5 methylation in actively transcribed tRNAs, in mouse and human tissues expressing and lacking NSun2 (Supplementary Fig S1A) (Blanco *et al*, 2011; Martinez *et al*, 2012). To identify actively transcribed tRNA genes, we first performed Pol III chromatin immunoprecipitation followed by DNA sequencing (ChIP-seq) and then validated tRNA expression by sequencing cDNA from total RNA (Supplementary Fig S1B). Finally, to determine NSun2 site-specific occurrence of cytosine-5 methylation in tRNA, we performed RNA bisulphite sequencing and miCLIP (Supplementary Fig S1B).

**Figure 1:**
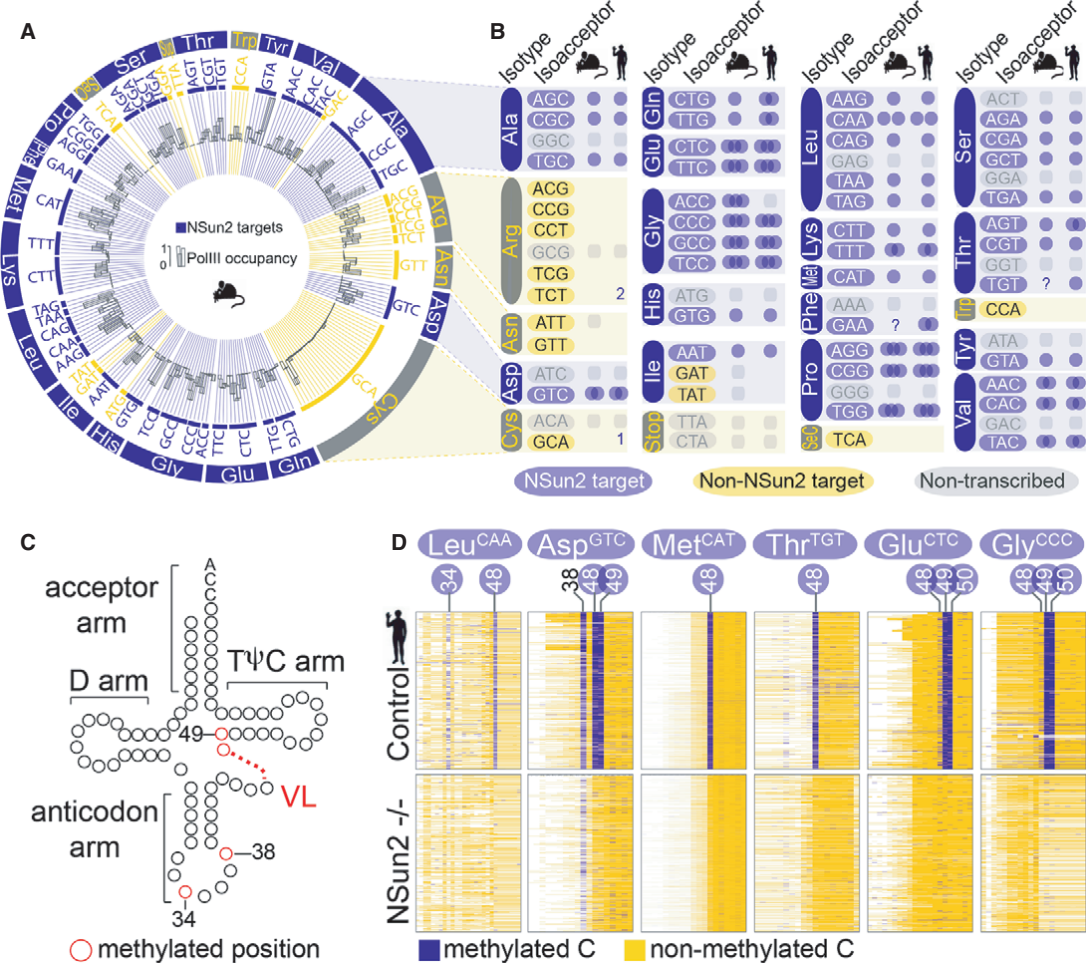
NSun2 methylates mouse and human tRNAs Circular plot of NSun2-targeted (blue bars) and not targeted or not expressed (yellow bars) tRNA genes discovered by RNA-seq and Pol III ChIP-seq in mouse skin. The outer ring shows tRNA isotypes, followed by isoacceptors (inner ring) and Pol III binding (black bars).Schematic summary of NSun2-methylated (blue) and not methylated (yellow) tRNA isoacceptors in mouse and human. Blue circles indicate the number of methylated cytosines by NSun2. Non-transcribed tRNA isoacceptors are marked in grey. 1,2: total number of methylated tRNA genes in that particular isoacceptor. ?: missing reads.tRNA secondary structure showing the acceptor arm, the D arm, the anticodon arm, the variable loop (VL) and the TΨC arm. The number of nucleosides in the variable loop (VL) vary (red dotted line). The position of known m^5^C-methylated cytosines in the anticodon loop (C34, 38) and the VL junction (C48, 49) is marked in red.Bisulphite-converted RNA to detect m^5^C in tRNAs in patient cells expressing (control) or lacking NSun2 (−/−). Shown are the sequence reads (rows) of methylated (blue) and unmethylated (yellow) cytosines (columns) in the indicated tRNAs. The positional number of each methylated cytosine is shown at the top. Blue circles mark NSun2-dependent methylation. C38 is methylated by Dnmt2. See also Supplementary Fig S1.

We identified a total of 252 actively transcribed tRNA genes in mouse skin, which highly overlapped (80–90%) with tRNA gene expression in mouse liver, muscle and testis (Supplementary Fig S1C and Supplementary Table S1) (Kutter *et al*, 2011). When NSun2 was deleted, the number of mature tRNAs was largely unaffected and Pol III occupancy to tRNAs remained unchanged (Supplementary Fig S1D and E; Supplementary Table S2). Bisulphite sequencing and miCLIP revealed that the vast majority (77%) of actively transcribed tRNAs were NSun2-specific substrates (Fig1A, and Supplementary Table S1). The 194 methylated tRNAs corresponded to 37 (out of 47) isoacceptor families and 16 (out of 21) isotype classes (Fig1A and B).

To investigate whether NSun2-targeted tRNAs were conserved between mouse and human, we compared from human skin fibroblasts of individuals carrying either a homozygous (−/−) or as control a heterozygous (+/−) loss-of-function mutation in the NSUN2 gene (Supplementary Fig S1A) (Martinez *et al*, 2012). Bisulphite sequencing identified 82% (324/391) of predicted tRNA genes were NSun2 methylation substrates, which highly overlapped with methylated tRNAs identified by miCLIP (245/324) and Aza-IP (223/324) (Supplementary Fig S1F) (Khoddami & Cairns, 2013). Similar to mouse, the methylated tRNAs grouped in 38 (out of 48) isoacceptor families and 16 (out of 21) isotype classes (Fig1B, and Supplementary Table S3). Thus, cytosine-5 methylation of tRNA is a highly conserved and common modification that can occur in up to 80% of all transcribed tRNAs in mouse and human.

### Site-specific cytosine-5 methylation is conserved

We next determined whether the occurrence of m^5^C in tRNAs was site-specific. tRNAs contain five conserved domains (Fig1C). Bisulphite sequencing confirmed robust cytosine-5 methylation in the anticodon loop at C34 (tRNA Leu^CAA^) and C38 (tRNAs Asp, Gly Val), and the intersection between the variable loop (VL) and the TΨC arm (VL junction) (48/49/50) (Fig1C and D, and Supplementary Fig S2A) (Motorin *et al*, 2010; Squires *et al*, 2012; Tuorto *et al*, 2012; Khoddami & Cairns, 2013). As previously reported, methylation at C34 and C48/49/50 was solely dependent on NSun2 (Fig1D) (Motorin & Grosjean, 1999; Brzezicha *et al*, 2006; Blanco *et al*, 2011; Martinez *et al*, 2012; Squires *et al*, 2012; Tuorto *et al*, 2012; Khoddami & Cairns, 2013). Methylation of C38 is mediated by Dnmt2 (Goll *et al*, 2006) and remained unchanged when NSun2 was deleted (Fig1D; Asp^GTC^).

**Figure 2:**
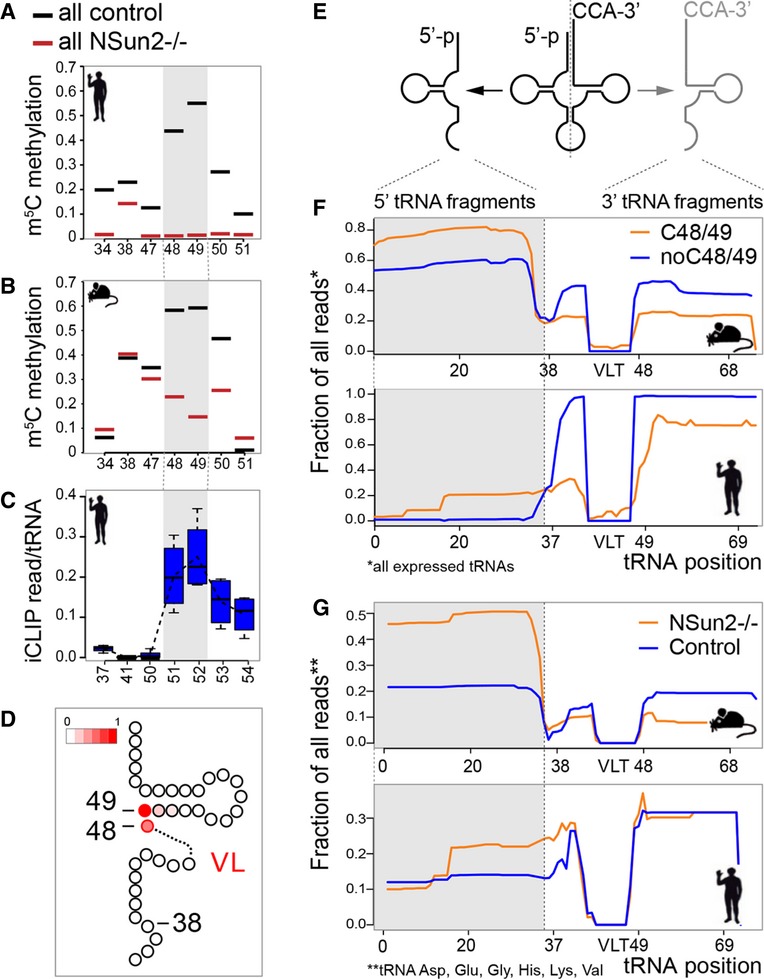
NSun2-targeted tRNAs accumulate as 5′ tRNA fragments A, B Frequency of m^5^C in all tRNAs in NSun2-expressing (control) and NSun2-lacking (−/−) human (A) and mouse (B) tRNAs. Grey boxes indicate cytosines 48 and 49.C Detection of NSun2-binding to cytosines in tRNAs using miCLIP method. Grey box indicates cytosines 48 and 49.D Scheme of 3′ half of a tRNA cloverleaf structure showing frequency of methylation at C48, 49 (red).E Scheme of cleaved tRNA through the anticodon loop.F Average fraction of 5′ (grey box) and 3′/CCA-tagged tRNA fragments using all expressed tRNAs with (orange line) and without (blue line) a cytosine at positions 48 and 49 in NSun2-expressing mouse (upper panel) and human (lower panel) samples.G Isotype-specific enrichment of 5′ tRNA fragments (grey box) in NSun2^−/−^ (orange line) versus respective control (blue line) in mouse (upper panel) and human (lower panel) samples. See also Supplementary Figs S2 and S3.

To determine whether there was a structural prevalence of specific cytosines to be methylated, we plotted the frequency of methylated cytosines in all mouse and human tRNAs (Fig2A and B; black line; Supplementary Fig S2B and C). Around 60% of all tRNAs carried a m^5^C modification at position 48 or 49 that was lost in the absence of NSun2 (−/−) in both human and mouse (Fig2A and B; grey box). A substantial fraction of the tRNAs carrying a cytosine at position 49 was simultaneously methylated at C48 or C50, demonstrating that m^5^C can occur at more than one cytosine within the VL of a tRNA (Supplementary Fig S2D and E). Notably, we find that more than 60% of tRNAs without a cytosine at position 49 were methylated at C48 instead (Supplementary Fig S2F and G), suggesting a conservation of cytosines at positions 48 and 49 among all tRNA genes. Methylation at C34 and 38 was rare and mostly undistinguishable from background noise (Fig2A and B, and Supplementary Fig S2B and C).

Using miCLIP, we further confirmed that in all tRNAs, the NSun2 cross-linked site corresponded to position 48 and 49 with highest frequency (Fig2C, and Supplementary Fig S2H). In human fibroblasts, we find the NSun2-tRNA cross-linked sites three bases downstream from the nearest cytosine (position +3) (Supplementary Fig S2I). Accordingly, cytosines C48 and 49 are represented by position 51 and 52 (Fig2C). Together, our data indicated a strong prevalence of the positions C48 and C49 within the VL junction of tRNAs to carry the m^5^C modification (Fig2D).

In summary, the vast majority of actively expressed tRNAs (> 80%) carried NSun2-mediated m^5^C modifications. Our data confirmed that NSun2-specific recognition and methylation in mouse and human was both sequence- and structure-specific.

### Non-methylated tRNAs are prone to accumulate as 5′ tRNA fragments

Stability of Asp^GTC^ and Gly^GCC^ tRNAs decreases when both cytosine-5 methylases Dnmt2 and NSun2 are absent in mice (Tuorto *et al*, 2012; Hussain *et al*, 2013c). In contrast, we found no evidence that loss of NSun2-specific methylation reduced abundances of specific mature tRNA isoacceptors (Supplementary Fig S3A). Since Pol III binding to tRNA genes was undistinguishable in wild-type and NSun2 knockout samples (Supplementary Fig S1D), we excluded the possibility that the loss of specific tRNA isoacceptors was compensated by transcription of alternative tRNA genes. We rather propose that general tRNA expression, processing and quality control are largely unaffected in the absence of NSun2.

Recent studies show that tRNAs can be cleaved through the anticodon loop into two short non-coding RNA fragments in yeast, plants and mammals (Fig2E) (Thompson *et al*, 2008). We detected a consistent enrichment of 5′ tRNA halves (˜35 nucleotides) in NSun2 knockout skin (Supplementary Fig S3B). Several lines of evidence indicated that the accumulated 5′ tRNA fragments specifically derived from NSun2-targeted tRNAs. We observed a higher frequency of 5′ tRNA fragments derived from tRNAs carrying a cytosine at 48/49 compared to tRNAs without a cytosine at these positions in mouse and human (Fig2F, and Supplementary Table S4). Although we only detected a modest increase in 5′ tRNA fragments when NSun2 was deleted (Supplementary Fig S3C), we identified a group of NSun2-targeted tRNA isotypes (Asp, Glu, Gly, His, Lys, Val), in which 5′ tRNA fragments were selectively enriched in the absence of NSun2 in mouse and human (Fig2G, and Supplementary Table S4). Thus, only specific sets of non-methylated tRNA were cleaved, yet the enriched isotype-specific 5′ tRNA fragments represented the vast majority (80%) of all detected fragments. Isotypes not methylated by NSun2 (i.e. Cys) showed no enrichment of 5′ tRNA halves in the absence of NSun2 (Supplementary Fig S3D).

To exclude a potential bias in our data set caused by compensation of loss of m^5^C by other chemical modifications that may cause an early stop of reverse transcription of RNA into cDNA (Alexandrov *et al*, 2006), we measured the occurrence of other chemical modifications in tRNAs by mass spectrometry in wild-type versus NSun2-depleted skin (Supplementary Fig S3E). Only the occurrence of m^5^C was significantly changed in the absence of NSun2 (Supplementary Fig S3E). We further confirmed the absence of any potential technical bias in our samples by plotting the mismatches between RNAseq data and the genomic reference that were the likely result of chemical modifications within tRNAs (Supplementary Fig S3F) (Findeiss *et al*, 2011). Other technical biases can result from the tRNA sequencing protocol itself. However, these errors are genotype independent and will affect all samples equally.

Our data show that loss of m^5^C at the variable loop of tRNAs increased the abundance of 5′ tRNA fragments and that tRNA fragmentation was the likely result of endonucleolytic cleavage of mature tRNAs through the anticodon loop.

### NSun2 and 5′ tRNA fragments co-localise with stress markers

Cleavage of tRNAs is a conserved response to several stress stimuli in eukaryotes (Thompson *et al*, 2008; Fu *et al*, 2009; Yamasaki *et al*, 2009; Emara *et al*, 2010), suggesting that the methylation activity of NSun2 may be inhibited in response to stress to trigger tRNA cleavage. However, RNA expression levels of NSun2 increased in response to UV radiation *in vitro* and *in vivo* (Supplementary Fig S4A and B), and tRNA cleavage also occurred in wild-type NSun2-expressing cells in response to oxidative stress (NaAsO_2_) (Supplementary Fig S4C). Therefore, we speculated that inhibition of NSun2 rather occurred on protein level.

**Figure 3:**
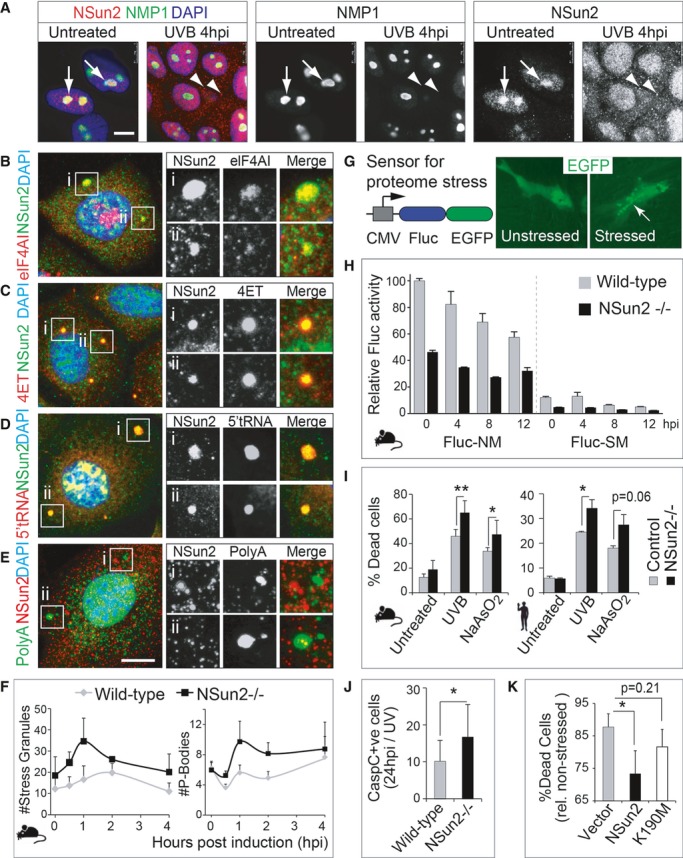
Loss of NSun2 enhances cellular stress responses A Immunofluorescence for NSun2 (red) and NMP1 (green) in untreated (left hand panels) and UV-radiated (right hand panels) human primary keratinocytes. Arrows indicate nucleoli, and arrowheads point to NSun2-positive cytoplasmic granules.B–E Co-localisation of NSun2 (green: B–D; red: E) with markers for SGs (eIFAI; red) (B), PBs (4ET; red) (C), 5′ tRNA halves (red) (D) or polyA RNA (green)-containing granules (E) in mouse primary keratinocytes after 4 h of exposure to UV. (i) and (ii) are magnified cytoplasmic granules for the respective stainings.F Automated counting of number of SGs (left hand panel) and PBs (right hand panel) per mouse cell after exposure to UV for the indicated hours. Error bars: SEM (*n* = 3 experiments; 1 experiment: all cells in 30 optical fields).G Schematic view of the proteome stress sensor Fluc-GFP construct (left hand panel) and cytoplasmic localisation of GFP-tagged Fluc construct (green) changes to protein aggregates after stress (right hand panel).H Luminescence activity of Fluc-NM (not mutated) and Fluc-SM (single mutation) stress reporters in wild-type and NSun2^−/−^ mouse keratinocytes upon UV exposure. Fluc luminescence is normalised to Renilla activity. Error bars, SD (*n* = 3).I Percentage of dead mouse (left hand panel) and human (right hand panel) primary cells in culture expressing (Control) or lacking NSun2 (−/−) 24 h after UV radiation and exposure to NaAsO_2_.J Quantification of cleaved caspase-3-positive cells in back skin of wild-type (WT) and NSun2 knockout (−/−) mice after 24 h of UV radiation.K Percentage of dead cells overexpressing an empty vector control (Vector), NSun2 or mutated NSun2 K190M after 24 h of UV treatment. Data information: In (I–K) error bars represent SD (*n *≥ 3). *P* < 0.05 (*) or *P* < 0.01 (**). Nuclei are counterstained with DAPI (blue) (A-E). Scale bar: 10 μm (A-E). hpi: hours post-induction. See also Supplementary Fig S4. Source data are available online for this figure.

To investigate how the enzymatic activity of NSun2 might be inhibited in response to stress, we examined the cellular localisation of NSun2 in primary human and mouse skin cultures exposed to UVB radiation. The vast majority of the NSun2 protein is found in the nucleoli, where tRNA methylation takes place (Fig3A; untreated; arrow) (Colonna & Kerr, 1980; Frye & Watt, 2006; Hussain *et al*, 2009). In response to UV radiation, however, NSun2 was excluded from the nucleoli and re-localised to the nucleoplasm and cytoplasmic granules (Fig3A; UVB 4 hpi, arrowheads), a process that was not due to nucleolar disassembly, since nucleolar markers such as NMP1 remained unaffected by UV exposure (Fig3A; NMP1). NSun2 re-localisation to the nucleoplasm and cytoplasm also occurred in response to NaAsO_2_ (Supplementary Fig S4D). These data suggested that under stress, NSun2-specific methylation of tRNAs at the nucleolus becomes inhibited by its relocation to cytoplasmic granules.

We next asked whether the cytoplasmic granules where NSun2 was found in response to stress represented stress granules (SGs) or processing bodies (PBs), both of which self-assemble in response to environmental stress (Anderson & Kedersha, 2008). In response to oxidative stress, the NSun2 protein only partly overlapped with markers for SGs (Fig3B; Supplementary Fig S4E) but co-localised with markers for PBs (Fig3C; Supplementary Fig S4F). The core proteins of PBs include proteins of the 5′ to 3′ decay machinery, nonsense-mediated decay pathway and RNA-induced silencing machinery (Anderson & Kedersha, 2006). tRNA fragments are also found in cytoplasmic granules but are not associated with polysomes, suggesting that they are not directly involved in translation (Reifur *et al*, 2012). Accordingly, we also find co-localisation of NSun2 with 5′ tRNA but not 3′ tRNA fragments or polyA RNAs (Fig3D and E; Supplementary Fig S4G), confirming that both tRNA methylation and protein translation are absent in NSun2-positive cytoplasmic granules.

### NSun2^−/−^ cells are more sensitive towards stress stimuli

Stress-induced tRNA fragments promote SGs assembly (Emara *et al*, 2010). We asked whether lack of NSun2 increased the number of SGs and PBs. In response to stress, the digitally counted number of both SGs and PBs was consistently higher in NSun2-depleted cells compared to wild-type controls (Fig3F). Both SGs and PBs self-assemble in response to stress-induced perturbations in translation, such as oxidative stress (Anderson & Kedersha, 2008). To maintain protein homoeostasis, cells typically respond to stress through global inhibition of translation accompanied by selective translation of survival proteins (Simpson & Ashe, 2012). To determine translational stress in the presence and absence of NSun2, we used as reporters destabilised versions of firefly luciferase (Fluc), whose luminescence activity reflects imbalances in cellular proteostasis in response to various stress stimuli (Fig3G) (Gupta *et al*, 2011). The GFP-tagged Fluc variants can further be used to measure insolubility and formation of protein aggregates (arrow) in response to stress (Fig3G; right hand panels) (Gupta *et al*, 2011).

As expected due to stress-induced translational repression, the destabilised version of Fluc (Fluc-SM) decreased in luminescence activity in wild-type and NSun2-depleted cells, and the stability of each construct was reduced in response to UV-induced stress (Fig3H) (Gupta *et al*, 2011). Importantly, the luminescence activity of NSun2^−/−^ cells was consistently lower compared to wild-type cells even in the absence of any stress stimulus (Fig3H; 0 hpi). We confirmed an imbalance in protein homoeostasis in human fibroblasts lacking NSun2 using the GFP-tagged Fluc variants and found a significant higher number of stressed cells after 8 h of incubation with NaAsO_2_ (Supplementary Fig S4H). We further confirmed that the higher sensitivity of NSun2^−/−^ cells to cellular stress resulted in an increase in apoptotic cells in both mouse and human skin cells in response to UVB radiation and oxidative stress (Fig3I).

NSun2-depleted cells are able to maintain normal skin homoeostasis (Blanco *et al*, 2011), but when we treated wild-type and NSun2^−/−^ mouse back skin with UV light, lack of NSun2 increased sensitivity towards external stress stimuli also *in vivo* (Supplementary Fig S4I). After 24 h of UV treatment, the number of apoptotic cells was higher in NSun2-depleted skin (Fig3J). To show that cellular survival after stress directly depended on NSun2 methyltransferase activity, we measured the percentage of dead cells in response to UV radiation in primary human keratinocytes overexpressing either wild-type or a mutant NSun2 construct (K190M), that is unable to methylate tRNA (Hussain *et al*, 2009). Overexpression of wild-type but not catalytically inactive NSun2 (K190M) significantly increased survival under stress compared to the empty vector control (Fig3K). We conclude that loss of NSun2-mediated RNA methylation sensitises cells to oxidative stress stimuli.

### Loss of NSun2 impairs survival of cortical, hippocampal and striatal neurons

**Figure 4:**
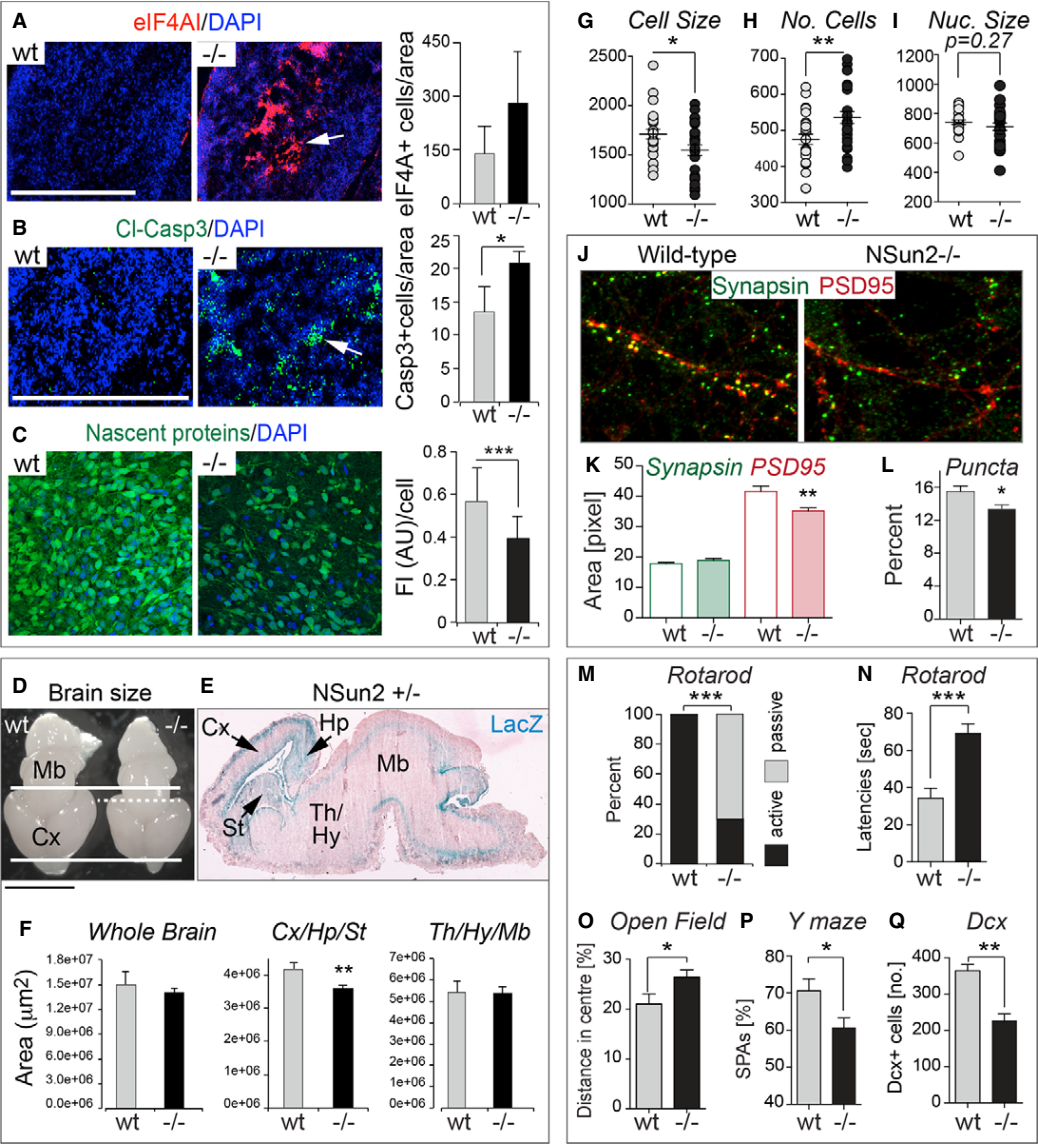
NSun2 deletion causes neuro-developmental defects in mice A, B Immunofluorescence staining for stress granules (eIF4AI, red) (A) and apoptosis (cleaved caspase-3, green) (B) in brain sagittal sections of E18.5 embryos. Arrows indicate brain areas stained with the indicated marker. Automated counting of number of stressed cells (eIF4AI^+^ cells) (A) or apoptotic cells (cleaved caspase-3^+^ cells) (B) per million μm^2^ in the whole brain is shown on the right. Error bars: SD (*n *≥ 3). Nuclei are counterstained with DAPI (blue).C Fluorescence labelling of nascent proteins (green) in cultured brain slices of E18.5 embryos incubated with OP-puromycin. Quantification of fluorescence intensity per cell is shown on the right. FI (AU): fluorescence intensity (arbitrary units). Error bars: SD (*n* ≥ 3 mice; 1 mouse: all cells in 10 optical fields). Nuclei are counterstained with DAPI (blue).D Brains of NSun2^−/−^ (−/−) and control (wt) E18.5 embryos. Thick lines delimit the rostrocaudal extent of the wt cerebral cortex (Cx). Dash line delimits the cortex of NSun2^−/−^ brain.E LacZ staining (blue) of a brain sagittal section of NSun2^+/−^ E18.5 embryo. Blue indicates NSun2 expression. Pink is eosin counterstaining. Arrows indicate highest NSun2 expression.F Areas of the whole brain, cortex, hippocampus and striatum (Cx/Hp/St), and thalamus, hypothalamus and midbrain (Th/Hy/Mb) measured from sagittal sections. Error bars: SD (*n *≥ 3).G–I Automated counting of cell size in pixels (G) and number of cells (H) of cortical neurons in NSun2^−/−^ (−/−) and control (wt) E18.5 embryos and nuclear size (I) measured as number of cells analysed per optical field. Error bars: SD (*n* ≥ 3 mice; 1 mouse: all cells in seven optical fields).J–L Analysis of synaptic puncta formation in cultured cortical neurons from NSun2^−/−^ (−/−) and wild-type (NSun2^+/+^, wt) E17.5 embryos. (J) Immunofluorescence of pre- (green) and post-synaptic puncta (red). (K) Automated area analysis of pre- (green) and post-synaptic puncta (red). (L) Percentage of functional synapses. Error bars: SD (*n* ≥ 3 mice; 1 mouse: all cells in seven optical fields).M–Q Behavioural differences in NSun2 ^−/−^ versus wild-type (wt) adult mice. (M) Latency to fall (active) or to stay (passive) on the rotarod. Shown is the percentage of animals with at least one passive rotation. Wt: *n* = 13; −/−: *n* = 26.N Mean latencies of three trials to fall on the rotarod (genotype *P* = 0.09, body mass *P* = 0.55; trial number *P* < 0.0001; linear mixed effects model). Wt: *n* = 13; −/−: *n* = 26.O Percentage of the distance travelled in the centre of the open field. Wt: *n* = 20; (−/−): *n* = 20. Error bar: SEM.P Percentage spontaneous alternations (% SPAs) in the Y maze using male wt and −/− mice (wt: *n* = 6; −/−: *n* = 10). Error bar: SEM.Q Number of doublecortin (DCX)^+^ cells in the hippocampal dentate gyrus in adult male mice. Error bar: SEM. Data information: *P < 0.05, **P < 0.01, ***P < 0.001. Scale bar: 0.5 cm (D), 500 μm (A, B). See also Supplementary Figs S5 and S6. Source data are available online for this figure.

In line with our data in cultured cells (Fig3), we also found increased expression of stress markers (eIF4AI) and cell death (cleaved caspase-3) in NSun2-deficient brains (Fig4A and B, and Supplementary Fig S5A and B). To confirm that global protein translation in the brain was affected in the absence of NSun2 *in vivo*, we cultured mouse embryonic brain slices in the presence of O-propargyl-puromycin (OP-puro), which is incorporated into nascent polypeptide chains and can be visualised by fluorescence microscopy (Liu *et al*, 2012). We digitally confirmed a reduction in protein translation in NSun2-deficient embryonic brains compared to wild-type controls (Fig4C).

The morphological consequence of increased cellular stress, cell death and lower protein translation was a significant reduction in brain size in NSun2 knockout embryos at E18.5 compared to wild-type littermates (Fig4D; Supplementary Fig S5C). The reduction in size and weight was specifically due to decreased areas of the cerebral cortex (Cx), hippocampus (Hp) and striatum (St) where NSun2 was highest expressed (Fig4E and F). Co-labelling studies confirmed that radial glial (GFAP^+^) and neuronal cells (Tbr1^+^; Dcx^+^) were equally affected by cell death (TUNEL^+^) (Supplementary Fig S5D–F).

Protein synthesis pathways are directly coupled to cell size (Lloyd, 2013). To test whether the reduction of NSun2^−/−^ brain sizes was solely dependent on neuronal cell death or also caused by altered cellular size, we digitally measured cortical and hippocampal neuron cell size by labelling their somata with a fluorescent Nissl stain. We found a reduction in neuronal cell size that was confirmed by a corresponding increase in cell number per area when NSun2 was deleted (Fig4G and H). Nuclei sizes remained unchanged (Fig4I).

Dysregulation of protein translation alters local protein synthesis, which is essential for synapse development and neuron maturation (Darnell, 2011). To test whether lack of NSun2 affected synapse formation, we isolated cortical and hippocampal neurons from wild-type and NSun2^−/−^ embryos and examined their gross level of synaptic puncta (Fig4J). While the levels of the pre-synaptic marker synapsin were similar in wild-type and NSun2^−/−^ neurons, we detected a reduction in the post-synaptic marker PSD95 (Fig4K), resulting in a decrease of functional synaptic puncta in the absence of NSun2 (Fig4L). Our data indicated that increase in cellular stress and dysregulation of protein synthesis affected maturation processes such as cell growth and synaptogenesis in NSun2-deficient mice. Neurodegenerative processes due to synaptic damage may in turn cause the neuronal loss in NSun2-deficient brains.

To test whether the observed developmental defects lead to aberrant brain function in adulthood, we screened NSun2^−/−^ adult mice for neurological defects. Depending on the mouse strain, homozygous deletion of NSun2 can be subviable (Supplementary Fig S6A) and occasionally adult NSun2^−/−^ mice show signs of ataxia (*not shown*). Surviving adult NSun2^−/−^ mice displayed a significant increase in passive rotations on the rotarod (Fig4M), a failure in test performance. Mean latencies to fall in the rotarod test were increased (Fig4N), what may be the consequence of the passive rotation behaviour and the reduced body mass. In the open field test, mice lacking NSun2 spent more time and travelled longer distances in the centre than wild-type controls (Fig4O, and Supplementary Fig S6B). Together, these tests indicate that loss of NSun2 altered motor coordination or balance and reduced adaptive anxiety related behaviours in a novel environment.

Because mutations in NSun2 can cause ID, we next subjected male NSun2^−/−^ mice to behavioural tests that involve cortical and hippocampal regions of the brain and allow the assessment of cognition. Using the Y maze spontaneous alternation test, we found a reduction of spontaneous alternations (% SPA) in male NSun2^−/−^ mice suggesting deficient spatial working memory (Fig4P). To evaluate recognition memory, we performed the social discrimination test, but did not find any differences (Supplementary Fig S6C). Notably, brain histological analyses of male NSun2^−/−^ mice revealed a significant decrease of Dcx-positive neurons in the dentate gyrus when compared to the wild-type controls (Fig4Q), confirming a reduced rate of hippocampal neurogenesis also in the adult brain (Couillard-Despres *et al*, 2005).

Our data indicated that loss of NSun2 impaired cortical, hippocampal and striatal expansion during development by triggering cellular stress responses and cell death.

### 5′ tRNA fragments are sufficient and required to induce cellular stress

**Figure 5:**
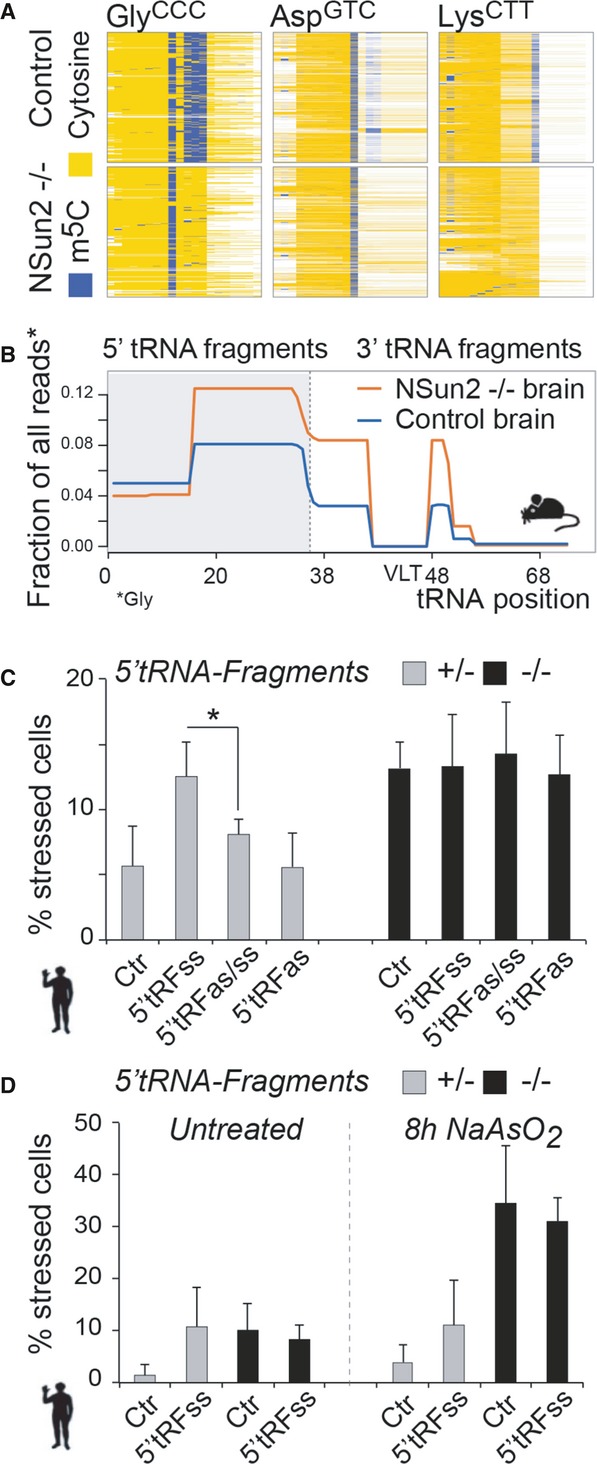
Isotype-specific enrichment of 5′ tRNA fragments in NSun2^−/−^ embryonic brains and induction of stress by 5′ tRNA fragments Bisulphite-converted RNA to detect m^5^C (blue columns) in tRNAs in the frontal lobe of brains from E18.5 embryos expressing (control) or lacking NSun2 (−/−).Isotype-specific enrichment of 5′ tRNA fragments (grey box) in NSun2^−/−^ (orange line) versus respective control (blue line) in E18.5 embryonic brains.Percentage of NSun2^+/−^ and NSun2^−/−^ stressed (with aggregated GFP) human fibroblasts in response to synthetic sense 5′ tRNA fragments (5′ tRFss) or sense and antisense 5′ tRNA fragments (ss, as).Stress response of untreated (left hand panel) and treated with NaAsO_2_ (right hand panel) NSun2^+/−^ and NSun2^−/−^ human fibroblasts to 5′ tRNA fragments. Data information: tRF: tRNA fragment; as: antisense; ss: synthetic sense; Ctr: mock treated (scrambled RNA). Error bars: SD (*n* = 3); *P < 0.1. See also Supplementary Fig S7. Source data are available online for this figure.

To confirm that cortical, hippocampal and striatal cell death was accompanied by methylation-dependent tRNA cleavage, we performed bisulphite sequencing and small RNA sequencing using RNA isolated from the frontal part of wild-type and NSun2^−/−^ brains. We confirmed highly frequent NSun2-mediated cytosine-5 tRNA methylation that was absent in knockout brains (Fig5A). We further identified glycine as the major NSun2-targeted tRNA isotype being cleaved, causing a selectively enrichment of 5′ tRNA fragments in NSun2-deficient brains (Fig5B).

To determine whether 5′ tRNA fragments are sufficient and required to induce cellular stress responses similar to deletion of NSun2, we transfected synthetic sense (ss) and antisense (as) 5′ and 3′ tRNA fragments (tRF) into human fibroblasts expressing (+/−) or lacking (−/−) NSun2 (Fig5C and D, and Supplementary Fig S7A). To measure stress, we quantified the number of cells with insoluble GFP-protein aggregates (GFP-tagged Fluc; Fig3G). We only detected an increase in the number of stressed cells when sense 5′ tRNA fragments were transduced into fibroblasts expressing NSun2 (+/−), an effect that was rescued by simultaneous transfection of sense (ss) and antisense (as) 5′ tRNA fragments (Fig5C; grey bars). The synthetic 5′ tRNA fragments failed to increase the number of NSun2^−/−^ stressed cells (Fig5C; black bars), which is likely due to the already elevated amounts of 5′ tRNA fragments and stimulated stress response. The number of stress-induced NSun2^+/−^ cells after 5′ tRNA fragment transfection was similar to control-transfected NSun2^−/−^ cells, indicating that 5′ tRNA fragments induced comparable stress levels to deletion of NSun2 (Fig5D; untreated). Interestingly, the stress response was not enhanced when 5′ tRNA transfected NSun2^+/−^ or NSun2^−/−^ fibroblasts were exposed to oxidative stress (Fig5D; 8 h NaAsO_2_). 3′ tRNA fragments failed to induce stress responses under any condition (Supplementary Fig S7A). Thus, the intracellular increase of synthetic or stress-induced 5′ tRNA fragments is sufficient to trigger cellular stress.

### tRNAs lacking cytosine-5 methylation exhibit increased affinity to angiogenin

So far, our data indicated that loss of NSun2 elevated stress responses and cell death as a consequence of increased 5′ tRNA fragmentation. Since the cleaved 5′ tRNA fragments derived from NSun2-targeted tRNAs, we speculated that m^5^C-methylation at the variable loop protected tRNAs from cleavage. The ribonuclease angiogenin mediates tRNA cleavage by targeting the anticodon loop in human cells (Fig2E) (Yamasaki *et al*, 2009). In *Drosophila*, Dnmt2-dependent m^5^C-methylation at the anticodon loop protects the tRNAs Asp^GTC^, Val^AAC^ and Gly^GCC^ from endonucleolytic cleavage (Schaefer *et al*, 2010). However, a similar protective role of cytosine-5 tRNA methylation in mammals has yet to be confirmed, and it was unknown whether NSun2-mediated methylation of tRNAs at the variable loop would provide protection from angiogenin-mediated cleavage.

**Figure 6:**
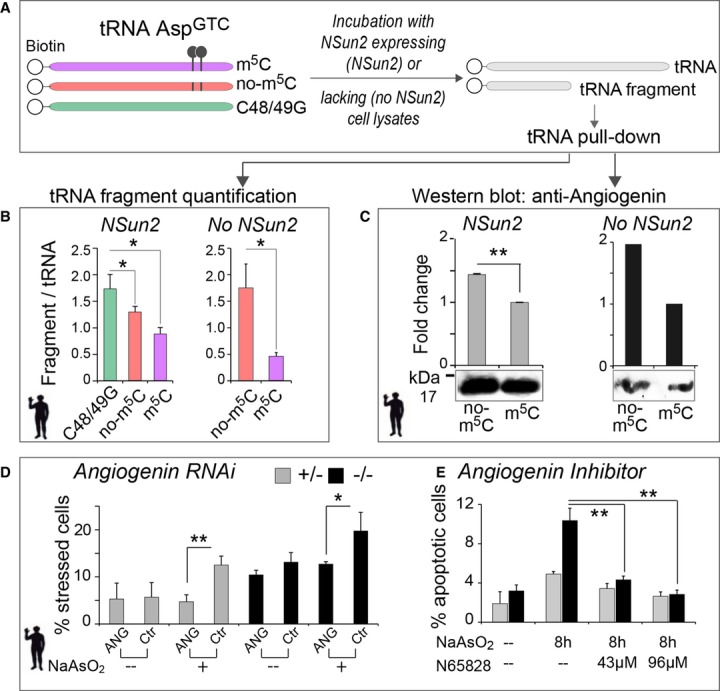
Cytosine-5 methylation protects from cleavage by angiogenin and angiogenin inhibition rescues elevated stress levels of NSun2^−/−^ cells A Schematic illustration of *in vitro* synthesised 5′ biotinylated tRNA Asp^GTC^ constructs (left hand panel) with (purple) or without m^5^C (red) at position 48 and 49, and a construct with substituted cytosines to guanosines (C48/49G; green). Synthetic tRNA constructs are incubated with NSun2 expressing (NSun2^+/−^, NSun2) or lacking (NSun2^−/−^, no NSun2) cell lysates to measure tRNA fragmentation and angiogenin binding.B Quantification of tRNA fragments pooled after incubation with cell lysates. Error bars: SD (*n* = 3).C Western blot (lower panel) and quantification (upper panel) of angiogenin bound to the indicated tRNA constructs after incubation with cell lysates. Pooled full-length tRNA was used for normalisation. Error bars: SD (*n* = 3).D, E Percentage of NSun2^+/−^ and NSun2^−/−^ stressed (D) or apoptotic (E) cells when angiogenin is inhibited by RNAi (D) or the small-molecule inhibitor N65828 (E). Cells are either treated for 8 h with NaAsO_2_ or untreated (–) as indicated. Data information: ANG: angiogenin RNAi; Ctr: mock treated (scrambled RNA). Error bars: SD (*n* = 3); *P < 0.1 and **P < 0.05. See also Supplementary Fig S8. Source data are available online for this figure.

To test whether the presence or absence of m^5^C at C48 and C49 directly determined the cleavage of human and mouse tRNAs by angiogenin, we generated four synthetic versions of 5′ biotin-labelled tRNA Asp^GTC^: (i) m^5^C-methylated at cytosines 48 and 49 (m^5^C) and (ii) non-methylated tRNA (no-m^5^C), as well as (iii) tRNA with cytosine substitution by guanine at position 48 alone (C48G) or (iv) substituted at positions 48 and 49 (C48/49G) (Fig6A, and Supplementary Fig S8A). Of note, all the *in vitro* synthesised tRNAs are not methylated at C38 and therefore can still be m^5^C-methylated at C38 in the presence of Dnmt2. We then incubated the synthetic tRNAs with cell lysates from NSun2^+/−^ (NSun2-expressing cells) (Fig6A; NSun2) or NSun2^−/−^ human fibroblasts (NSun2 lacking cells) (Fig6A; no NSun2). After incubation with cell lysates, we measured the ratio of purified cleaved 5′ ends versus full-length tRNA (Fig6A and B, and Supplementary Fig S8B). Enrichment of 5′ tRNA fragments correlated with loss of methylation at C48/49 (Fig6B; left hand panel; Supplementary Fig S8B–D). We confirmed increased cleavage of non-methylated overmethylated tRNA after incubation with NSun2^−/−^ lysates (Fig6B; right hand panel; Supplementary Fig S8E). To further determine that m^5^C-methylation at C48/49 shielded tRNA from angiogenin binding, we analysed the amount of angiogenin bound to the purified tRNA. Although the quantities of full-length C48/49G and non-methylated tRNAs were reduced (Fig6B), the constructs bound angiogenin with higher affinity (Fig6C, and Supplementary Fig S8F), indicating that methylated C48/49 protected the tRNA from binding to and being cleaved by angiogenin. We obtained the same results using methylated and non-methylated tRNA Lys^CTT^ (Supplementary Fig S8G and H). tRNA Lys^CTT^ is not a Dnmt2 substrate.

### 5′ tRNAs-induced stress can be rescued by inhibition of angiogenin

To test whether cleavage of tRNAs lacking m^5^C at position 48 and 49 was solely dependent on angiogenin or included additional endonucleases, we inhibited angiogenin by RNAi and the small-molecule inhibitor N65828 (Kao *et al*, 2002). Reduction of angiogenin mRNA using RNAi significantly decreased the number of stressed cells both in the presence and absence of NSun2 after NaAsO_2_ exposure (Fig6D, and Supplementary Fig S8I). Similarly, incubation of NSun2-depleted cells with N65828 inhibited cleavage of tRNA and significantly reduced the number of apoptotic cells under oxidative stress to a level comparable to control cells (Fig6E, and Supplementary Fig S8J). However, simultaneous transfection of 5′ tRNA-F and incubation with N65828 failed to protect from stress, confirming that 5′ tRNA-F act downstream of angiogenin (Supplementary Fig S8K).

**Figure 7:**
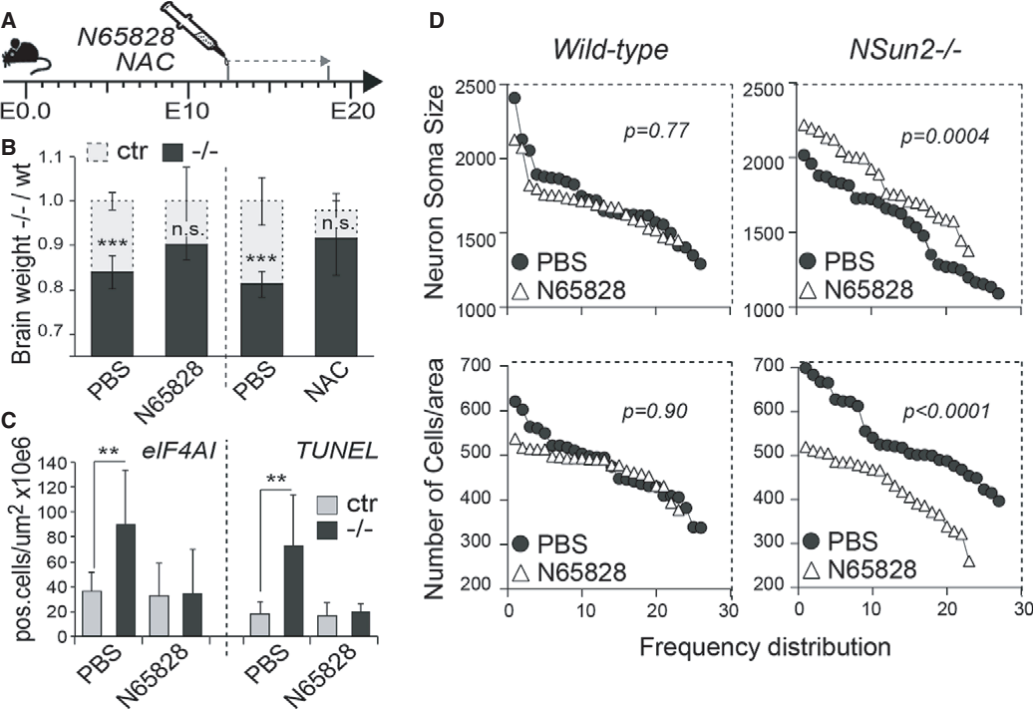
Angiogenin inhibition rescues the deleterious effects of loss of NSun2 during neuro-development *in vivo* A Injection schedule of pregnant mouse females with N65828 (angiogenin inhibitor) and NAC along embryonic development.B–D Rescue of neuro-developmental defects in NSun2^−/−^ mice *in vivo*. (B) Brain weight fold change of NSun2^−/−^ (−/−, black bars) versus wild-type (wt, grey bars) embryos after N65828 and NAC treatment as indicated in (A). Control embryos were treated with PBS. (C) Number of stressed cells (eIF4AI^+^ cells) (left hand panel) or apoptotic cells (TUNEL^+^ cells) (right hand panel) per million μm^2^ in the frontal brain lobe of wild-type (Ctr) and NSun2^−/−^ (−/−) embryos after treatment. (D) Frequency distribution of size (pixels) (top panels) and number of cell counts per area (bottom panels) of cortical neurons from wild-type and NSun2^−/−^ E18.5 embryos after treatment. Error bars: SD (*n *≥ 3). *P < 0.1, **P < 0.05. and ***P < 0.001 (B, C). Source data are available online for this figure.

To test whether inhibition of angiogenin rescued the deleterious effects of loss of NSun2 in the brain *in vivo*, we injected pregnant NSun2^+/−^ females with the angiogenin inhibitor N65828 or as a control, the antioxidant N-acetylcysteine (NAC) to inhibit oxidative stress responses (Fig7A). Both treatments rescued the brain sizes of NSun2^−/−^ embryos. Only NSun2^−/−^ embryos that received either N65828 or NAC showed no significant difference in brain size when compared to their wild-type littermates (Fig7B). The increased stress response (elF4AI) and elevated cell death (TUNEL) were also rescued after treatment with either drug (Fig7C, and Supplementary Fig S9A). Furthermore, treatment with N65828 significantly increased neuronal cell size and decreased the number of cells per area in embryonic NSun2^−/−^ brains (Fig7D; right hand panels) but did not affect cell size or number in wild-type brains (Fig7D; left hand panels). We obtained similar results when NAC was administered (Supplementary Fig S9B). Thus, the reduced size of NSun2^−/−^ brains is caused by a combination of increased cell death and decreased cellular size. Together, our data show that NSun2 promotes survival of neuronal cells in response to cellular stress and is essential for the full maturation of neurons during development.

Together, our data provide the first mechanistic explanation why patients lacking functional NSun2 display symptoms of neuro-developmental disorders. We suggest that accumulation of cleaved 5′ tRNA fragments induce stress responses in the absence of any known external stress stimuli leading to cell death. The tRNA cleavage induced by loss of NSun2 is strictly dependent on angiogenin, suggesting that angiogenin inhibitors may be suitable drugs to alleviate at least some of the symptoms found in patients with loss-of-function mutations in NSun2.

## Discussion

Here, we show that post-transcriptional methylation of tRNA is a major upstream regulator determining the intracellular concentration and identity of 5′ tRNA-derived small RNA fragments. tRNA hypo-methylation caused by inhibition or lack of NSun2 allows angiogenin-mediated tRNA cleavage and leads to the accumulation of 5′ tRNA fragments. These 5′ tRNA fragments activate stress-response pathways leading to reduced protein translation rates, decreased cell size and increased cell death *in vitro* and *in vivo* causing a syndromic disorder characterised by growth and neuro-developmental deficiencies in mice and human. Both inhibition of tRNA cleavage and oxidative stress pathways during mouse embryogenesis abrogate the stress-induced pathways and revert cell size and survival to normal. Thus, we identified the first mechanistic link between loss-of-function of the *NSUN2* gene and the neuro-developmental disorder in humans.

### tRNA and tRNA modifications in human disease

tRNAs are a fundamental component of the translation machinery and genetic mutations that affect mRNA translation commonly contribute to human cognitive and behavioural diseases (Scheper *et al*, 2007; Darnell, 2011). tRNAs are heavily modified post-transcriptionally and mutations in a range of tRNA modifying enzymes have been linked to human neurological diseases (Torres *et al*, 2014). Even tRNA modifications itself have been linked to human disease (Suzuki *et al*, 2001). However, the molecular mechanism linking tRNA modification to human diseases is largely unknown. We now show that tRNAs lacking cytosine-5 methylation are prone to be cleaved by angiogenin, and altered tRNA cleavage due to mutations in angiogenin is also linked to neurodegenerative diseases, such as amyotrophic lateral sclerosis and Parkinson's (van Es *et al*, 2011; Skorupa *et al*, 2012).

### Cytosine-5 methylation, tRNA cleavage and cellular stress

Angiogenin-induced fragmentation of tRNAs to inhibit translation is a common response to various stress conditions (Gold *et al*, 1963; Thompson *et al*, 2008; Fu *et al*, 2009; Yamasaki *et al*, 2009; Ivanov *et al*, 2011; Abbasi-Moheb *et al*, 2012; Saikia *et al*, 2012). Although cellular factors regulating tRNA cleavage are largely unknown, cytosine-5 methylation recently emerged as one important determinant of tRNA fate in response to stress (Schaefer *et al*, 2010; Chan *et al*, 2012). Levels of m^5^C in tRNAs change in response to oxidative stress in yeast (Chan *et al*, 2010), and Dnmt2-mediated cytosine-5 methylation can protect the tRNAs Asp^GTC^, Val^AAC^ and Gly^GCC^ from endonucleolytic cleavage in *Drosophila* (Schaefer *et al*, 2010). Although NSun2 and Dnmt2 show some functional redundancy *in vivo*, the proteins show little homology on protein level and the distinct methylated cytosines are non-overlapping and enzyme-specific (Tuorto *et al*, 2012). Therefore, the function of NSun2-mediated tRNA cytosine-5 methylation in response to oxidative stress in mammals remained unknown. We now show that in contrast to Dnmt2, NSun2 methylated the vast majority of RNA polymerase III transcribed tRNAs (> 80%) *in vivo*. RNA bisulphite sequencing is one of the three recently developed methods to globally map RNA methylation (Hussain *et al*, 2013a). The two other methods, miCLIP and Aza-IP, are based on RNA immunoprecipitation approaches followed by next-generation sequencing (Hussain *et al*, 2013b; Khoddami & Cairns, 2013). The NSun2-specific methylated tRNAs identified by bisulphite sequencing highly overlapped with those identified by miCLIP (this study) and Aza-IP (Khoddami & Cairns, 2013). Only RNA bisulphite sequencing determined that all tRNAs carrying a cytosine at position 48 or 49 are specifically methylated by NSun2.

Cleaved 5′ tRNA fragments accumulate in stressed cells (Thompson *et al*, 2008; Fu *et al*, 2009; Yamasaki *et al*, 2009; Emara *et al*, 2010). We show that loss of NSun2 not only elevated isotype-specific 5′ tRNA fragmentation but also increased cellular stress. We find that deposition of m^5^C at the variable loop protects tRNAs from binding to and cleavage by angiogenin, confirming that cytosine-5 methylation can inhibit tRNA–protein interactions (Chen *et al*, 1993). High expression levels of NSun2 in the developing mouse brain or in skin after UV exposure indicate a protective role of RNA methylation. Together, these data reveal cytosine-5 methylation in tRNAs as essential regulator to evade unnecessary cleavage of mature and functional tRNAs during the stress response. Furthermore, we reveal cytosine-5 methylation at the variable loop of tRNAs as the main upstream regulator of angiogenin-dependent tRNA binding and cleavage because inhibition of angiogenin in the absence of NSun2 rescued the reduced protein translation, cell size as well as stress-induced cell death *in vitro* and *in vivo*.

### Stress-induced RNA cleavage and human disease

Stress-induced RNA cleavage and release of ribonucleases into the cytosol have been linked to several human diseases but mainly to cancer (Thompson & Parker, 2009). Emerging evidence also indicates a potential role of elevated oxidative stress in neuro-developmental diseases such as autism, Rett syndrome and Down syndrome (De Felice *et al*, 2012; Lintas *et al*, 2012; Essa *et al*, 2013). Similarly, loss-of-function of the kinase CLP1 causes neurological diseases in mouse and human due to aberrant accumulation of tRNA fragments caused by impaired pre-tRNA processing and increased cell sensitivity to oxidative stress (Hanada *et al*, 2013 Schaffer *et al*, 2014; Karaca *et al*, 2014).

Loss-of-function mutations in angiogenin have also been associated with neurological diseases, supporting the hypothesis is that angiogenin rather exerts a neuro-protective role (Greenway *et al*, 2006; Steidinger *et al*, 2011). Our data challenge this view because inhibition of angiogenin in the absence of tRNA methylation promotes cellular survival. These controversial findings may be explained by the tightly regulated substrate recognition of angiogenin through post-transcriptional modifications and its inability to cleave methylated tRNAs.

### Molecular implications of 5′ tRNA fragment accumulation

The biological role of stress-induced tRNA cleavage is not to degrade mis-processed or hypo-modified tRNAs or to reduce the pool of mature tRNAs (Lee & Collins, 2005; Thompson *et al*, 2008; Yamasaki *et al*, 2009), and the fate of the two tRNA fragments after processing is diverse. In some cases, both tRNA halves or only the 3′ fragments are stable in the cell (Haiser *et al*, 2008; Lee *et al*, 2009; Haussecker *et al*, 2010; Zywicki *et al*, 2012). In contrast, depletion of NSun2 or external stress conditions primarily induces accumulation of 5′ tRNA fragments (Cole *et al*, 2009; Yamasaki *et al*, 2009; Emara *et al*, 2010; Garcia-Silva *et al*, 2010; Ivanov *et al*, 2011; Gebetsberger *et al*, 2012; Zywicki *et al*, 2012; Hanada *et al*, 2013). 5′ tRNA halves are specifically produced to repress translation in response to stress by displacing translation initiation and elongation factors from mRNAs or by interfering with efficient transpeptidation (Spriggs *et al*, 2010; Ivanov *et al*, 2011; Gebetsberger *et al*, 2012; Sobala & Hutvagner, 2013).

Lower protein translation rates may directly be responsible for the reduced neural cell size and impaired formation of synapses in NSun2 knockout brains (Darnell, 2011; Lloyd, 2013). The reduced protein translation in the absence of NSun2 may explain also the general growth retardation observed in mice and human lacking the NSun2 protein (Blanco *et al*, 2011; Abbasi-Moheb *et al*, 2012; Khan *et al*, 2012; Martinez *et al*, 2012; Tuorto *et al*, 2012). We demonstrate that impaired protein translation, decreased neuronal cell size as well as increased cell death in NSun2 knockout brains are directly caused by stress-induced tRNA cleavage because all of these major phenotypes in the mouse brain can be reverted through administration of an inhibitor of angiogenin or oxidative stress. Our data suggest the use of small-molecule inhibitors of angiogenin to protect from stress-induced cell death and to alleviate some of the symptoms found in patients.

In summary, our study is the first to functionally link the loss of cytosine-5 methylation in tRNAs to accumulation of short tRNA-derived non-coding RNAs in human diseases. Our data suggest that aberrant post-transcriptional methylation of RNA may be a novel and important contributor to human diseases.

## Material and Methods

### Ethics statement and mouse lines

All mouse husbandry and experiments were carried out according to the local ethics committee under the terms of the UK Home Office licence PPL80/2231 and PPL/2619. NSun2^−/−^ mice carrying a Gene Trap in intron 8 of the NSUN2 gene (GGTC-clone ID: D014D11) have been described (Blanco *et al*, 2011). Behavioural test were performed at the German Mouse Clinic according to standard protocols.

### Cell lines and cell culture

Patient dermal fibroblasts (NSun2^−/−^) and control dermal fibroblasts (NSun2^+/−^) were grown in MEM (Invitrogen) supplemented with 20% foetal bovine serum (FBS) and antibiotics in a humidified atmosphere at 37°C and 5% CO_2_ as described previously (Martinez *et al*, 2012). Mouse keratinocytes were isolated and cultured as described previously (Blanco *et al*, 2011).

### Antibodies and reagents

The following primary antibodies were used: rabbit polyclonal anti-human NSun2 (1:1,000; Meta) (Frye & Watt, 2006), rabbit polyclonal CUK-1079-A anti-mouse NSun2 (1:1,000; Covalab) (Blanco *et al*, 2011), mouse monoclonal anti-NMP1 (1:500; Sigma, B0556, clone FC82291), mouse monoclonal anti-angiogenin (1:200; Abcam, ab10600, clone 14017.7), goat polyclonal anti-eIF4AI (1:200; N-19) (Santa Cruz, sc-14211), goat polyclonal anti-4ET (1:200; E-18) (Santa Cruz, sc-13454), goat polyclonal anti-SK1 (1:200; A-13) (Santa Cruz, sc-17991), goat polyclonal anti-eIF3η (1:200; A-20) (Santa Cruz, sc-16378), rabbit polyclonal anti-cleaved caspase-3 (1:100; Cell Signaling, #9664), mouse anti-PSD95 (1:200; Thermo Scientific), rabbit antisynapsin (1:1,000; Synaptic Systems), mouse anti-GFAP (1:200; Millipore), rabbit anti-Tbr1 (1:500, Abcam), rabbit anti-Dcx (1:100, Abcam).

NaAsO_2_ was used at 200 μM (Sigma). The angiogenin small-molecule inhibitor N65828 (8-amino-5-(4′-hydroxybiphenyl-4-ylazo)naphthalene-2-sulphonate) was obtained from the National Cancer Institute (http://dtp.cancer.gov). NAC was purchased from Sigma and O-propargyl-puromycin (OP-puromycin) from Medchem Source LLP.

### Chromatin immunoprecipitation (ChIP) sequencing library preparation

Pol III ChIP-seq was performed and analysed as reported (Kutter *et al*, 2011). Briefly, total mouse back skin was fixed in 1% formaldehyde (v/v), lysed, sonicated and then incubated with antibodies recognising antigen POLR3A, the RPC1/155 subunit of Pol III. The immunoprecipitated material was end-repaired, A-tailed, ligated to the sequencing adapters, amplified by 18 cycles of PCR and size-selected (200–300 base pairs). Each experiment used mouse skin of 3- to 3.5-week-old male mice pooled together. A minimum of two independent biological replicates from wild-type and one for NSun2^−/−^ animals were used for the analyses. The circular histogram was generated using Circos software (http://circos.ca/).

### tRNA sequencing library preparation

The libraries were generated from mouse snap-frozen back skin isolated from 3.5-week-old male mice, frontal lobe of E18.5 embryonic mouse brains and human dermal fibroblast. At least four independent biological replicates were carried out for all the experiments. Total RNA was extracted using Trizol reagents (Invitrogen) from snap-frozen material and treated with DNase (Turbo DNase, Ambion). Ribosomal RNA was removed using Ribo-zero (Epicentre, Illumina). The remaining RNA fraction was gel size-selected or with the MirVana Isolation Kit (Invitrogen) for 20- to 200-base-paired RNAs. tRNAs were then de-aminoacylated in 0.1 M Tris–HCl pH 9.0 and 1 mM EDTA for 30 min at 37°C. tRNA libraries were performed according to TruSeq Small RNA Preparation Kit (Illumina) instructions. Briefly, 3′ adenylated and 5′ phosphorylated adapters suitable for Illumina RNA sequencing were ligated to 300 ng of RNA obtained after de-aminoacylation. RNA was reverse-transcribed at 50°C for 1 h (SuperScript III cDNA synthesis kit, Invitrogen), followed by 16-cycle PCR amplification with Phusion DNA polymerase (Thermo scientific) and size-selected (140–210 base pairs) before sequencing.

### Bisulphite sequencing library preparation

Experiments were done from snap-frozen mouse back skin isolated from 3.5-week-old male mice and human dermal fibroblast. At least four independent biological replicates were carried out. Total RNA was extracted and DNase and Ribo-zero treated. The remaining RNA fraction was bisulphite-converted as followed; about 2 μg of RNA was mixed in 70 μl of 40% sodium bisulphite pH 5.0 and DNA protection buffer (EpiTect Bisulfite Kit, Qiagen). The reaction mixture was incubated for four cycles of 5 min at 70°C followed by 1 h at 60°C and then desalted with Micro Bio-spin 6 chromatography columns (Bio-Rad). RNA was desulphonated by adding an equal volume of 1 M Tris (pH 9.0) to the reaction mixture and incubated for 1 h at 37°C, followed by ethanol precipitation.

The bisulphite-converted RNA quality and concentration was then assessed on a Bioanalyzer 2100 RNA nano-chip (Agilent). About 120 ng of bisulphite-converted RNA was used to generate Bisulfite-seq libraries. Bs-Seq libraries were performed according to TruSeq Small RNA preparation kit (Illumina). Since the sample was already sufficiently fragmented by the bisulphite-conversion reaction (about 50–120 nt), the size selection step prior to adaptor hybridisation and ligation was not performed. First 2′,3′-cyclic phosphate and 5′-hydroxyl termini produced during the bisulphite/desulphonation reaction were end-repaired with T4 PNK and Spermidine (New England Biolabs). RNA adapters suitable for Illumina sequencing were then ligated, reverse-transcribed at 50°C for 1 h with SuperScript III and 1 mM of each dNTP (SuperScript III cDNA synthesis kit, Invitrogen) followed by 18-cycle PCR amplification. The amplified product was not size-selected before sequencing.

### miCLIP

The myc-tagged NSun2 C271A mutated construct (Hussain *et al*, 2009) or an empty vector control were transfected into human fibroblasts using an Amaxa nucleofection kit (Lonza), and cells were harvested 48 h later. miCLIP was subsequently performed as described previously (Hussain *et al*, 2013b).

### Illumina sequencing

After passing quality control on a Bioanalyzer 2100 DNA chip (Agilent), all libraries were sequenced on the Illumina Genome Analyzer II (single-ended and 100-bp read length) and post-processed using the standard GA pipeline software v1.4 (Illumina).

### Pol III ChIP-seq data analysis

In the mouse reference genome (UCSC NCBI37/mm9), 3,282 candidate tRNA genes were predicted with tRNAscan-SE (http://lowelab.ucsc.edu/tRNAscan-SE) including genes with an “undetermined” isoacceptor family; loci marked as pseudogenes were discarded. Pol III ChIP-seq reads were aligned to the reference genome using Bowtie (Langmead *et al*, 2009). Two mismatches and at most 500 multiple matches were tolerated, that is, specifically the Bowtie options “-m 500 -v2 -best -strata” were used. Reads that matched at multiple gene regions were distributed proportionally to the fraction of uniquely matching reads at these regions as described (Kutter *et al*, 2011).

### tRNA-seq data analysis

Adapters were removed, and the 20–200 nt fragments were aligned to the mouse and human reference genomes (UCSC mm9 and GRCh37/hg19) with the options “-m 500 -v2 -best -strata”, and multiply matching reads were distributed as described for Pol II ChIP-seq reads. To account for the addition of CCA 3′-ends to mature tRNAs, unaligned reads were trimmed of CCA[CCA] ends and realigned using the same options. Annotations were conducted based on the gene loci predicted by tRNAscan-SE in the mouse and based on previously predicted tRNA genes from GtRNAdb (http://lowelab.ucsc.edu/GtRNAdb) in human. Reads with CCA[CCA] ends that covered at least 90% of the tRNA gene were considered mature tRNAs. For fragment aligning at tRNA genes, reads that exceeded the gene start or end by more than 10% were discarded. For all mature tRNAs and tRNA fragments, counts were normalised and differential abundance of the fragments was evaluated with the Bioconductor/R package DESeq (Anders & Huber, 2010). For the analysis of short fragments of length 25–40 nt, no mismatches were allowed at the tRNA gene start positions and only 3′ fragments with post-transcriptionally modified CCA ends derived from mature tRNAs were included. To structurally align the tRNAs consistently, we determined the first paired nucleoside in the TΨC arm as position 49 in all our analyses (Kim *et al*, 1974).

### tRNA sequence variation analysis

Only tRNA-seq and Pol III ChIP-seq reads that mapped uniquely to the mouse reference genome with at most two mismatches were considered. The mismatches for both tRNA-seq and Pol III ChIP-seq reads at each position in the tRNA genes were counted. All tRNA genes were aligned according to their secondary structure, and mismatch counts for each gene were mapped to the corresponding position in the tRNA structural alignment using custom scripts. Average counts over all tRNA genes with at least 100 counts are shown.

### Bisulphite-seq analysis

Fastq files were mapped against all known human transcripts from the Ensembl v68 database (assembly GRCh37) using the Bismark program (Krueger & Andrews, 2011). Bismark transforms sequence reads into bisulphite-converted (C-to-T) and reverse read (G-to-A) versions before they are aligned to a similarly processed reference. Bismark takes sequence reads that produce a unique best alignment and compares these to the normal genomic sequence, and the methylation state of all cytosine positions in the read is inferred. For mouse, the exact same pipeline was used, the only difference was the ensembl assembly was GRCm38 and the version number was 67.

The alignment files were sorted using SAMtools, and the aligned reads were annotated using HTseq count (http://www-huber.embl.de/users/anders/HTSeq/). Using custom scripts, the position of each read was determined in regard to the gene. This was then converted to a matrix with a −1/+1 determining if a m^5^C was at a position in regard to a gene. In order to filter for high-confidence candidates, genes with < 10 reads aligned were discarded. A heatmap was generated using the program matrix2png from this matrix (http://www.chibi.ubc.ca/matrix2png/).

Reads annotated as belonging to tRNAs were compared to the structural alignment from the GtRNAdb database (gtrnadb.ucsc.edu). These structural alignments are generated by aligning tRNA sequences against domain-specific tRNA covariance models with the use of COVE (covariance models of RNA secondary structure). The number of 5-methylcytosines at each position in the structural alignment was calculated, and this percentage was plotted using the R statistical package. In order to filter for high-confidence candidates, reads with > 30% unconverted cytosines were discarded as were tRNAs with < 10 reads aligned.

### UV light exposure and NaAsO_2_ treatment of cells

Cells at 80% confluence were exposed to 80–120 J/m^2^ of UV light in a CL-1000 Ultraviolet Crosslinker (UVP). Briefly, media was removed before exposure and immediately replaced by warm fresh media. Cells were then incubated for the indicated time points before sample collection. Cells at 80% were incubated in media containing 200 μM NaAsO_2_ for the indicated time points. When longer than 2 h, media containing NaAsO_2_ was washed and replaced by warm fresh media before sample collection.

### Mouse UV light exposure

Back skin of 7-week-old mouse males was shaved 24 h before UV light exposure. For UV exposure, mice were restrained and only the shaved back area was exposed to UV light. Mice were then exposed into 2.5 kJ/m^2^ for about 5 min according to the UK Home Office terms an Ultraviolet Irradiation System (Tyler Research Corporation). Animals were then monitored and kept for 24 or 48 h before sample collection.

### Cell immunofluorescence and fluorescence *in situ* hybridisation (FISH)

Co-localisation of NSun2 with stress granules or processing bodies was performed as reported (Kedersha & Anderson, 2007). Briefly, cells were fixed for 15 min with 4% paraformaldehyde at room temperature, followed by 10 min −20°C methanol incubation. Cells were then blocked for 1 h with 5% normal horse serum at room temperature. Primary antibodies were incubated overnight at 4°C with the appropriate antibody dilution indicated in the antibodies section. Before secondary antibody incubation cells were washed three times for 10 min in PBS. Secondary antibodies (Alexa Fluor-conjugated, Invitrogen) were added at a dilution of 1:500 for 1 h at room temperature together with DAPI to label nuclei. Cells were finally washed as previously and mounted with Mowiol 4-88 (Calbiochem.).

*In situ* hybridisation to detect co-localisation with tRNA or poly(A) RNA with NSun2 was performed essentially as described previously (Kedersha *et al*, 1999) with some modifications. In brief, cells were fixed in 2% paraformaldehyde in PBS for 10 min, permeabilised with −20°C methanol for 10 min and washed twice in 2× SSC. To prevent folding and secondary structure, formation of the DNA oligo probe and the tRNAs to be detected were performed FISH under denaturing conditions, by heating the probes at 95°C for 5 min and the slides at 75°C for 5 min just prior to the hybridisation. Hybridisation was performed in a humid chamber at 43°C overnight in a 4× SSC buffer containing 0.1% Triton X-100 and 1 ng/μl of the DNA probe. The following Alexa Fluor-conjugated DNA oligo probes (Invitrogen) were used: 5′ end of AspGTC tRNA Alexa Fluor 555-conjugated 5′-GGG GAT ACT TAC CAC TAT ACT AAC GAG GA, 3′ end of AspGTC tRNA Alexa Fluor 488-conjugated 5′-CTC CCC GTC GGG GAA TT and a 50-mer OligodT Alexa Fluor 488-conjugated. Cells were washed in 4× SSC and then probed with anti-mouse NSun2 antibody (Covalab, 1:1,000) in 2× SSC containing 0.1% Triton X-100. After a 1-h incubation at room temperature, cells were again washed in 4× SSC and subjected to a final incubation with a solution containing 1:1,000 dilution of anti-rabbit IgG Alexa Fluor 594 or 488-conjugated (Invitrogen), and DAPI dye in 2× SSC/0.1% Triton X-100 for 1 h. Then, cells were sequentially washed in 4× SSC and 2× SSC, and mounted. Confocal images were acquired on a Leica TCS SP5 confocal microscope. All the images were processed with Photoshop CS5 (Adobe) software.

### Analysis of stress granules and processing bodies

Mouse keratinocytes in culture were first exposed to UV light as described before and fixed after the indicated time points. Cells were then stained with anti-eIF4AI antibodies to detect SGs and anti-4ET antibodies to detect PBs as described previously in cell immunofluorescence section. 30 images of each time point were automatically acquired using an Olympus IX80 microscope and a DP50 camera or Zeiss AxioImager, AxioCam MRm camera, AxioVision software at a 40× magnification. Number and size of SGs and PBs per cell were analysed using CellProfiler cell image analysis software (http://www.cellprofiler.org/). At least three independent experiments were carried out for each time point. Data were analysed using Microsoft Office Excel 2007 software (Microsoft).

### Cellular stress assessment assays

Cells were transiently transfected with DNA plasmids as follows: human and mouse cells were seeded in 24-well plates 24 h before transfection and grown in normal conditions. At the time of transfection, cells were at 70% confluence. Primary mouse keratinocytes were transfected with Attractene Transfection Reagent (Qiagen), according to their recommendations. Primary human fibroblasts were transfected with XtremeGENE DNA Transfection Reagent (Roche) according to the manual. Cells were transduced with the reporter constructs FlucNM-GFP (or FlucWT-GFP), FlucSM(R188Q)-GFP or FlucDM(R188Q, R261Q)-GFP kindly provided by Dr. S. Raychaudhuri (Gupta *et al*, 2011), and when required cells were also co-transfected with pRL (Renilla luciferase) (Promega) as transfection control. Cells were fixed to assess GFP aggregation, or luciferase activity was measured 24 h after the transfection. NaAsO_2_ was added to the media at the indicated time points before sample collection.

For co-transfection of human fibroblasts with the reporter constructs and single-stranded RNA oligos (or tRNA fragments), cells were treated as follows: first cells were transfected with DNA plasmids as indicated before using XtremeGENE. Then, cells were left recovering for 16 h and transfected with tRNAs using DharmaFECT I Transfection Reagent (Thermo Scientific), following the manufacturer recommended RNA concentrations and recommended protocol conditions. The following tRNA fragments synthetised by Dharmacon/Thermo Scientific were used: 5′Asp^GTC^tRNA-F synthetic sense (ss): 5′ UCC UCG UUA GUA UAG UGG UGA GUA UCC CCG CCU GUC; 3′Asp^GTC^tRNA-F synthetic sense (ss): 5′ CGC GGG AGA CCG GGG UUC GAU UCC CCG ACG GGG AG; antisense-5′Asp^GTC^tRNA-F (as): 5′ GAC AGG CGG GGA UAC UCA CCA CUA UAC UAA CGA GGA. AllStars Neg. siRNA AF 647 (Qiagen) was used as RNA negative control. When N65828 was used, it was added to the media 24 h before the DNA/XtremeGENE transfection. When NaAsO_2_ was used, it was added to the media from 1 to 8 h before sample collection. Cells were fixed 8 h after tRNA transfection.

For co-transfection of human fibroblasts with the reporter constructs and angiogenin RNAi, cells were treated as follows: 24 h after seeding, cells were transfected with Human ANG (283) siGENOME SMART pool (M-011206-01-0005, Dharmacon/Thermo Scientific) with DharmaFECT I Transfection Reagent as indicated before. AllStars Neg. siRNA AF 647 (Qiagen) was used as a scramble RNA control and to assess RNAi transfection. Cells were left recovering for 12 h and transfected with the reporter DNA constructs with XtremeGENE DNA Transfection Reagent (Roche). Cells were fixed 12–16 h after XtremeGENE transfection. NaAsO_2_ was added to the media from 1 to 8 h before sample collection.

Luciferase activity was measured using the Dual-Luciferase Reporter Assay System (Promega) on Glomax (Promega). To assess GFP aggregates, cells were fixed with 4% PFA for 10 min at room temperature. GFP aggregation was assessed using an Olympus IX80 microscope under 40× magnification. All transfected cells with and without GFP aggregates were counted. Each experiment was carried out in triplicates.

### Culture of human keratinocytes and retroviral infection

Human keratinocytes from healthy donors were kindly provided by Dr. S. Aznar-Benitah. Cells were grown on mitomycin C-treated J2-3T3 feeder cells on collagen type I (BD Biosciences)-coated plates (BD Falcon). Human keratinocytes were grown in FAD media (one part Ham's F12, three parts Dulbecco's modified Eagle's medium, 18 mM adenine and 0.05 mM calcium) supplemented with 10% FCS and a cocktail of 0.5 μg/ml of hydrocortisone, 5 μg/ml insulin, 10^−10^ M cholera enterotoxin and 10 ng/ml epidermal growth factor (HICE cocktail) and maintained in a humidified atmosphere at 37°C and 5% CO_2_. Keratinocytes were infected with pBabe empty vector, pBabe-NSun2(Misu) and pBabe-NSun2 (Misu)(K190M) constructs as described previously (Hussain *et al*, 2009).

### Apoptosis and survival assessment assays

To assess cell death by flow cytometry, cells were first incubated with NaAsO_2_ and N65828 for the indicated time points or UV light irradiated and left them recovered for the indicated time points. Cells were then shortly trypsinised and disaggregated by gentle pipetting. Trypsin was inactivated by adding media containing FCS. The cells were pelleted and re-suspended in binding buffer and stained for Annexin V (Annexin V Apoptosis detection kit, eBioscience) according to manufacturer's recommendations. Flow cytometry analysis was carried out on a CyAn ADP analyzer (Dako Cytomation), and data were processed in FlowJo.

### Tissue preparation and staining

Skin samples were fixed and embedded in paraffin using standard protocols. E18.5 embryonic brains were fixed with 4% PFA for 6 h and embedded in 7.5% sucrose/10% gelatin and frozen. H&E staining were carried out according to standard protocols. Immunostaining and LacZ staining were carried out as described before (Blanco *et al*, 2011). Primary antibodies used were described in the antibodies section. Secondary antibodies (Alexa Fluor-conjugated, Invitrogen) were added at a dilution of 1:500 for 1 h at room temperature together with DAPI to label nuclei. TUNEL staining was performed using DeadEnd™ Fluorometric TUNEL System (Promega). White field images were acquired using an Olympus IX80 microscope and a DP50 camera. Confocal images were acquired on a Leica TCS SP5 confocal microscope. All the images were processed with Photoshop CS5 (Adobe) software.

### RNA extraction and quantitative RT–PCR (QPCR)

Total RNA from cells or tissues was prepared using Trizol reagent (Invitrogen) according to the manufacturer's instructions. Double-stranded cDNA was generated from 1 μg total RNA using Superscript III First-Strand Synthesis kit (Invitrogen) and random hexamer primers (Promega). At least three technical replicates were analysed. Real-time PCR amplification and analysis was conducted using StepOne Real-Time PCR System (Applied Biosystems). The standard amplification protocol was used with pre-designed probe sets and TaqMan Fast Universal PCR Master Mix (2×) (Applied Biosystems). Probe sets Hs04195574_sH and Mm00520224_m1 were used to amplify human angiogenin and mouse NSun2, respectively. GAPDH expression (4326317E) was used to normalise samples using the ΔCt method.

### Northern blotting

10 to 20 μg RNA samples was loaded into a 15% TBE-urea gel, run and transferred to a Nylon neutral Hybond-NX membrane (GE Healthcare) for 90 min at 15 V in a semi-dry electrophoretic transfer cell unit (Biorad). Membranes were cross-linked for 2 h at 60°C with EDC solution (1-ethyl-3-(3-dimethylaminopropyl) carbodiimide) according to Kim *et al* (2010). Blots were pre-hybridised at 37°C for at least 30 min in ULTRAhyb solution (Ambion), followed by overnight probed hybridisation in ULTRAhyb at 37°C. 20 pmol of single-stranded DNA probes were 5′-end radiolabeled with ^32^P-γ-ATP (Perkin Elmer) and T4 PNK (Enzymatics) for 1 h at 37°C, followed by column purification (Illustra Microspin G-25 columns, GE Healthcare), denatured at 95°C for 5 min and added to the hybridisation solution. Membranes were then washed twice with low stringent buffer (0.1× SSC, 0.1% SDS) at 37°C for 15 min and then washed twice with high stringent buffer (2× SSC, 0.1% SDS) at 37°C for 5 min. Membranes were dried and exposed to X-ray films (Kodak). The following DNA probes were used (Sigma): mouse HisGTG-5′ end: CTA ACC ACT ATA CGA TCA CGG C; mouse and human GlyCCC-5′ end: GAT ACC ACT ACA CCA GCG GCG C.

### Mouse embryonic brain dissection, size measurement and cell counting

Mouse embryos were collected at E18.5. Brains were dissected out and fixed in 4% PFA for a minimum of 6 h. Embryonic brain weight was measured after fixation. Embryonic brains were then embedded in 7.5% sucrose/10% gelatin and frozen. Stainings were performed as described previously. Measurement of brain areas and number of cleaved caspase-3-positive, TUNEL-positive and eIF4AI-positive cells were calculated automatically using cell image analysis software Volocity. To measure neuron cell and nuclear size, brain sections were stained with NeuroTrace reagent (Life Technologies) and DAPI. Images were analysed automatically using Volocity. At least three independent experiments (embryos) were carried out for each genotype, and ten images per mouse were analysed.

### Labelling of nascent proteins in brain slices

Mouse embryos were collected at E18.5. Brains were quickly dissected out and embedded in 15% of low-melting agar to then superglue them onto a block for cutting. 200-μm fresh brain slices were cut using a Leica VT1000 A vibratome. Slices were quickly transferred to oxygenated slicing medium and allowed to settle for an hour prior to labelling. OP-puromycin at 50 μM was added to the slicing medium for 1 h according to Liu *et al* (2012). Brain slices were fixed with 4% PFA for 1 h and washed twice in TBS. After washing, the slices were stained using Click-iT® Cell Reaction Buffer Kit (Life Technologies). Brain slices not incubated with OP-puromycin but stained were used as negative controls. The tissue slices were counterstained with DAPI, mounted in standard mounting media and were then imaged by Leica TCS SP5 confocal microscope. The fluorescent intensity of individual cells was quantified automatically using CellProfiler software (http://www.cellprofiler.org/). At least three independent experiments (embryos) were carried out for each genotype, and ten images per mouse were analysed.

### Synaptic puncta analysis

Cortical and hippocampal neuron cultures were set from E17.5 mouse embryos according to Beaudoin *et al* (2012). Cells were maintained in culture for 1–2 weeks in Neurobasal medium (Life Technologies, w/o glutamine and w/o Phenol Red) supplemented with 200 mM B27 (Life Technologies), 2 mM glutamine, 0.3% glucose and 37.5 mM NaCl. Cells were fixed in 2% PFA and 15% glucose and stained for the indicated antibodies. Images were acquired with Leica TCS SP5 confocal microscope. Post- and pre-synaptic puncta were analysed using ImageJ software according to Ippolito and Eroglu (2010). At least three independent experiments (cultures) were carried out for each genotype, and ten images per culture were analysed. Functional puncta were calculated as the number of overlapping post- and pre-synaptic puncta normalised from the total number of synaptic punta.

### tRNA *in vitro* cleavage assay

One mg of BioMag-Streptavidin beads (Qiagen, cat 311711) was washed according to manufacturer's instructions and incubated with 100 pmol of 5′ biotinylated tRNA in BioMag Binding buffer for 30 min/RT. Cell extracts were prepared 2 h after NaAsO_2_ treatment in order to have internalised angiogenin. Cells were lysed in buffer containing or 1% NP-40, 25 mM Tris–HCl at pH 7.4, 30 mM NaCl, 10 mM MgCl_2_ and Complete Mini EDTA-free protease inhibitor (Roche). The lysate was then incubated for 30 min at room temperature and centrifuged for 20 min at maximum speed. After washing, beads were incubated with 0.5 ml cell extracts for 30 min to 1 h at 4°C on a rotating wheel. Beads were washed twice in the washing buffer recommended by the manufacturer, and tRNAs were eluted from beads for 30 min at room temperature in TE buffer pH 9.5. Full-length tRNA and tRNA fragment quality and quantity were assessed on a 2100 Bioanalyzer RNA Nanochip (Agilent).

The following biotinylated tRNAs (Thermo Scientific) were used: Asp^GTC^-WT: 5′UCC UCG UUA GUA UAG UGG UUA GUA UCC CCG CCU GUC ACG CGG GAG ACC GGG GUU CAA UUC CCC GAC GGG GAG CCA; m^5^C-Asp^GTC^-WT: 5′UCC UCG UUA GUA UAG UGG UUA GUA UCC CCG CCU GUC ACG CGG GAG Am^5^Cm^5^C GGG GUU CAA UUC CCC GAC GGG GAG CCA; Asp^GTC^-C48G: 5′UCC UCG UUA GUA UAG UGG UUA GUA UCC CCG CCU GUC ACG CGG GAG AGC GGG GUU CAA UUC CCC GAC GGG GAG CCA; Asp^GTC^-C48/49G: 5′UCC UCG UUA GUA UAG UGG UUA GUA UCC CCG CCU GUC ACG CGG GAG AGG GGG GUU CAA UUC CCC GAC GGG GAG CCA; m^5^C-Lys^CTT^-WT: 5′GCC CGG CUA GCU CAG UCG GUA GAG CAU GAG ACU CUU AAU CUC AGG GUm^5^C GUG GGU UCG AGC CCC ACG UUG GGC GCC A; Lys^CTT^-C48G: 5′GCC CGG CUA GCU CAG UCG GUA GAG CAU GAG ACU CUU AAU CUC AGG GUG GUG GGU UCG AGC CCC ACG UUG GGC GCC A.

### Angiogenin-binding assay

A protocol to detect angiogenin bound to tRNA has been modified from Castello *et al* (2012). Pellets from two 15-cm dishes were lysed in 2–3 ml of lysis/binding buffer [100 mM Tris–HCl, pH 7.5, 500 mM LiCl, 10 mM EDTA pH 8.0, 0.5% lithium-dodecylsulphate, 5 mM DTT, Complete Mini EDTA-free protease inhibitor (Roche)] and homogenised using a narrow gauge needle (0.4 mm diameter). One miligram of BioMag-Streptavidin beads was prepared as described above and added to 2 ml of cell extracts. Beads were incubated for 2 h at room temperature on a rotating wheel. Beads were washed twice in 5 ml buffer 1 containing 50 mM Tris–HCl, pH 7.5, 250 mM LiCl, 10 mM EDTA pH 8.0, 0.2% lithium-dodecylsulphate, 5 mM DTT, for 30 min at room temperature, followed by three washing steps in 5 ml washing buffer 2 (50 mM Tris–HCl, pH 7.5, 140 mM LiCl, 2 mM EDTA pH 8.0, 0.5% NP-40, 0.5 mM DTT). Protein–tRNA complexes were heat eluted from beads in SDS loading buffer for 5 min at 80°C and load in a 15% SDS–polyacrylamide gel. Proteins were transferred to a 0.2-μm-pore nitrocellulose membrane (Genscript Corporation). A mouse anti-angiogenin antibody (Abcam) was used at 1:250. Blots were incubated overnight at 4°C with primary antibodies, washed and incubated with the appropriate HRP-conjugated secondary antibodies (GE Healthcare). The chemiluminescent signal was detected using the ECL Plus Detection System (GE Healthcare). Densitometry was analysed with ImageJ software. tRNA bound to the magnetic beads was measured on a 2100 Bioanalyzer (Agilent) and used as loading control to normalise angiogenin binding. The 5′ biotinylated tRNAs used were described in the previous section.

### NAC and N65828 administration

For *in vitro* experiments, N65828 was added at concentration of 96 μM (or otherwise indicated) (Kao *et al*, 2002) and NAC was added at 50 μM to the culture medium. For *in vivo* administration, N65828 (400 μg/ml in PBS at pH 7.4) was subcutaneously injected every second day into pregnant NSun2^+/−^ female mice at a dose of 2.5 mg/kg. NAC, freshly made NAC (10 mg/ml in PBS at pH 7.4), was subcutaneously injected daily into pregnant NSun2^+/−^ mice at a dose of 67 mg/kg. Pregnant mice were treated from embryonic stages E12.5 until E18.5.

### Statistical analysis

If not otherwise stated, the significance of quantitative data was tested using the unpaired, two-tailed Student's *t*-test.

### Accession codes

All mouse sequencing data are available under the accession number GSE44746. All human sequencing data are available on dbGAP (phs000645.v1.p1).
