# Supplementary material for: Aberrant methylation of tRNAs links cellular stress to neuro-developmental disorders
Source: EMBO J. 2014 Jul 25;33(18):2020–39. doi: 10.15252/embj.201489282 (PMC4195770; doi:10.15252/embj.201489282)
Supplement: Supplementary file 1 [file embj0033-2020-sd1.pdf]

# Supplementary Figure 1

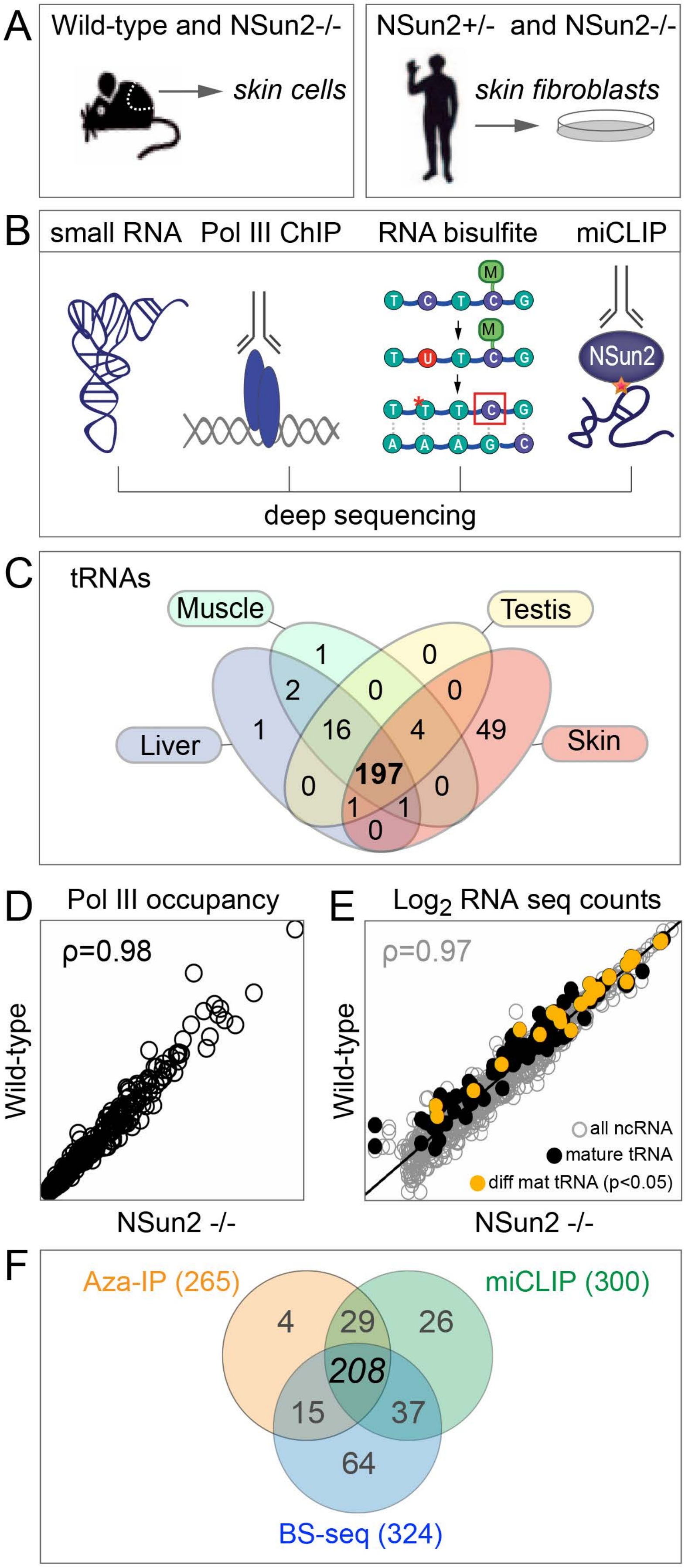

**Figure S1.** Identification of NSun2 methylated tRNAs in mouse and human tissues. (A) Back skin from wild-type and NSun2 -/- mice and primary cultured human skin fibroblasts from NSun2 -/- patients and NSun2 +/- healthy donors were collected. (B) Tissues and cells collected in (A) were flash-frozen to permit direct tRNA sequencing and RNA sequencing of bisulfite converted RNA, or treated with formaldehyde to cross-link protein-DNA contacts to allow RNA Polymerase III (Pol III) ChIP reactions and further sequencing of Pol III bound DNA molecules. NSun2-RNA complexes were immnuprecipitated (miCLIP) followed by RNA sequencing to detect NSun2 bound RNA molecules (C) Venn diagram of all common tRNA genes bound by Pol III in indicated mouse tissues. tRNA genes expressed in liver, muscle and testis were obtained from (Kutter et al., 2011). (D) Pol III occupancy at tRNA gene loci in wild-type and NSun2 -/- back skin. (E) Expression ($log_2$ normalized reads) of all non-coding (nc) RNAs (grey circles), full-length mature tRNAs (black circles) and differentially abundant tRNAs ($p<0.05$) (orange circles) in wild-type and NSun2 -/- back skin. ρ: pearson's correlation coefficient (D,E). (F) Venn diagram of NSun2-methylated tRNA genes found by bisulfite sequencing (BS-seq) and miCLIP (this study) and Aza-IP (Khoddami and Cairns, 2013).

# Supplementary Figure 2

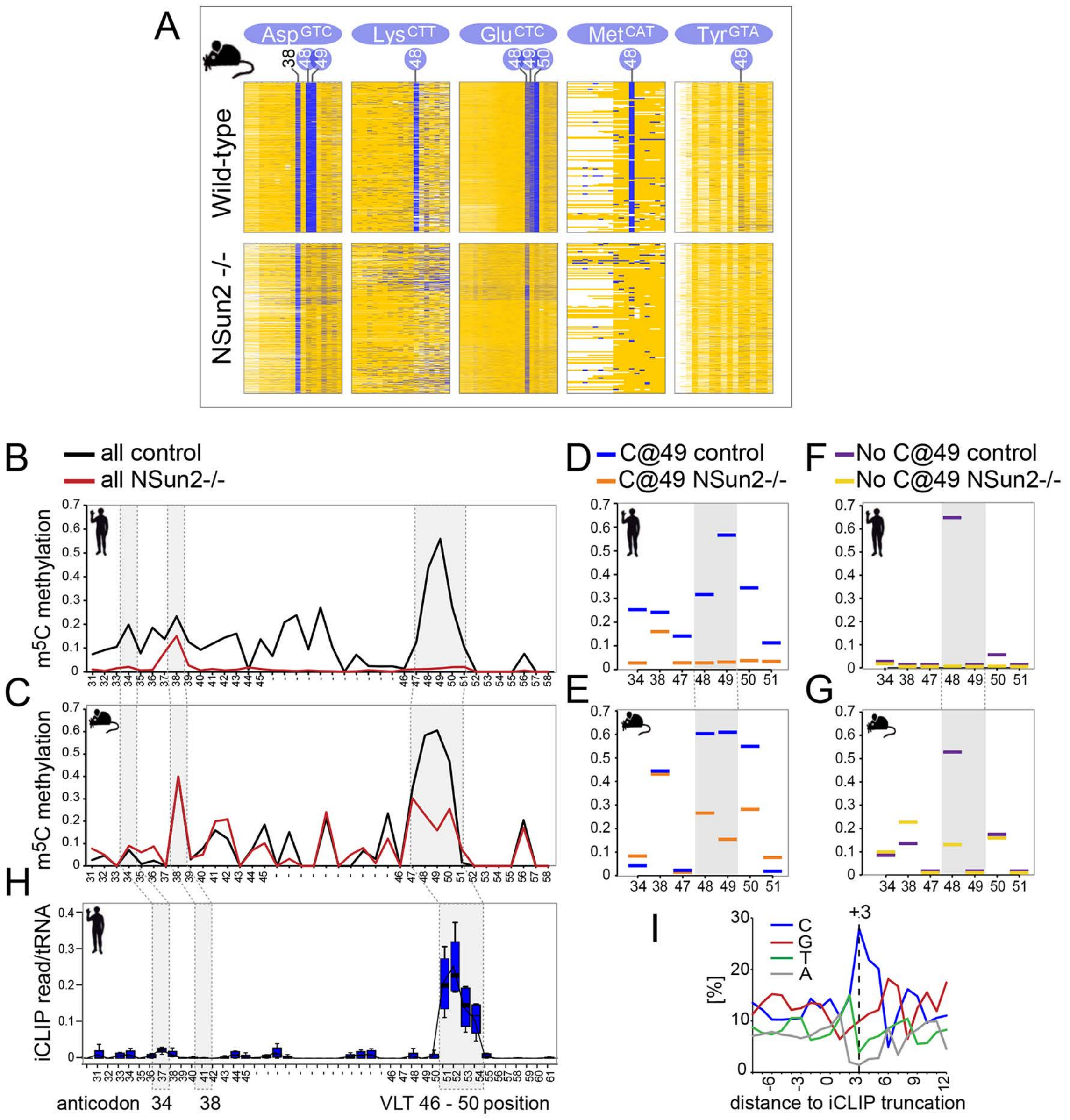


**Figure S2.** NSun2 preferentially methylates cytosines 48 and 49. (A) RNA bisulfite-sequencing heatmaps of NSun2-methylated tRNAs in wild-type (upper panels) and NSun2 -/- (lower panels) mouse skin. Shown are sequence reads (rows) of methylated (blue) and un-methylated (yellow) cytosines (columns) of the indicated tRNA transcripts. Blue circles mark NSun2-dependent methylation. (B-C) Frequency of $m^5C$ in all tRNAs along the tRNA length in NSun2-expressing (control) and NSun2-lacking (-/-) human (A) and mouse (B) tRNAs. (D-G) Frequency of $m^5C$ for the indicated cytosines in tRNAs with a cytosine at position 49 (D,E) and tRNA without a cytosine at position 49 (F,G) in NSun2-expressing (control) and NSun2-lacking (-/-) human (D,F) and mouse (E,G) tRNAs. (H) Frequency of NSun2-miCLIP reads terminating at the indicated tRNA nucleoside in +/- human fibroblasts. Grey boxes depict $m^5C$ tRNA methylated positions (B-H). (I) Frequency of NSun2-crosslinked cytosine at position +3.

# Supplementary Figure 3

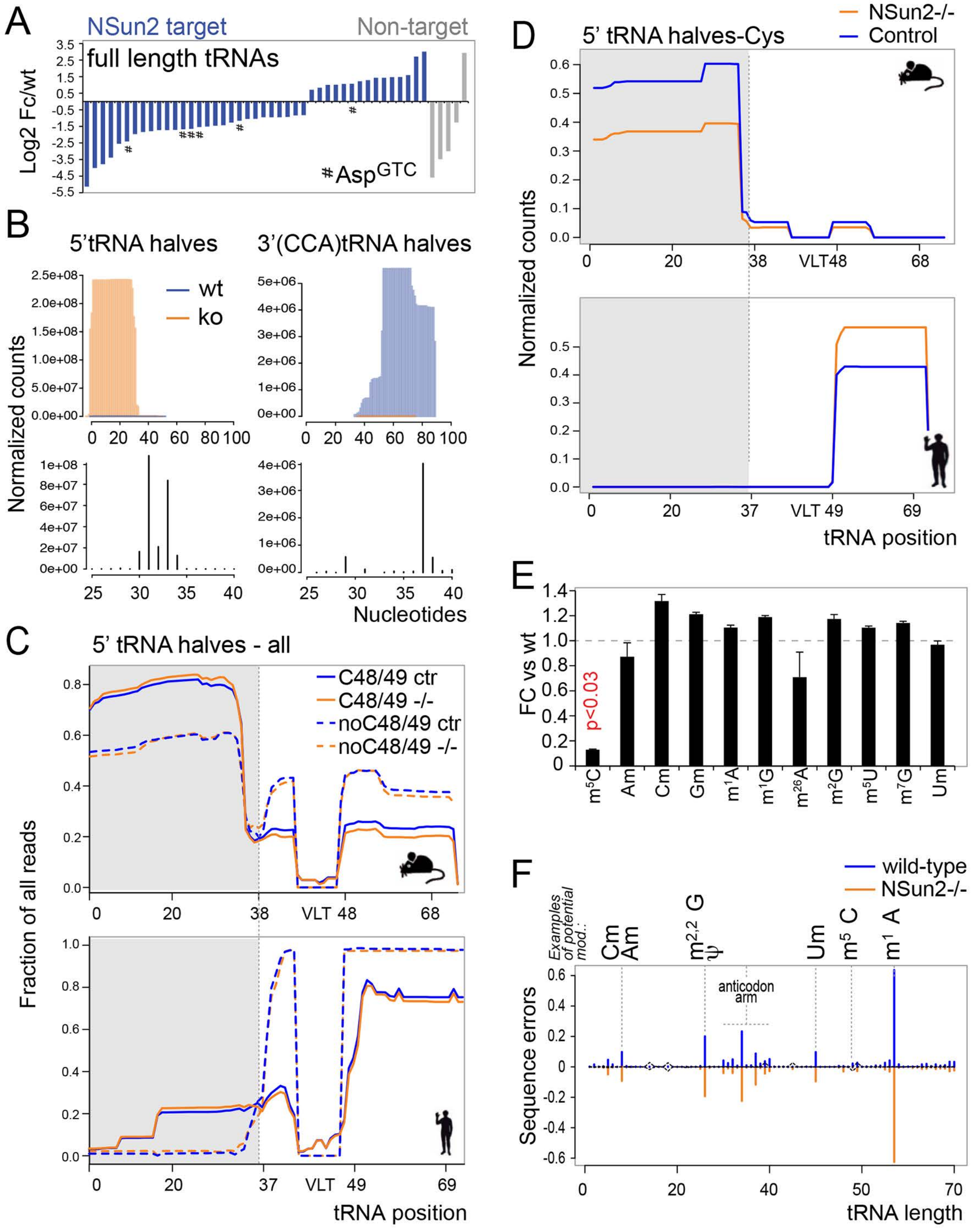

**Figure S3.** Accumulation of 5' tRNA halves and exclusion of bias in the datasets. (A) Full-length tRNA expression values ($log_2$ fold change; $p<0.05$) of NSun2-methylated (blue) and non-methylated (grey) tRNAs in NSun2 -/- versus wild-type mouse skin. Hash indicates tRNA genes encoding for the $Asp^{GTC}$ isoacceptor. (B) Normalized counts (upper panels) and average length (lower panels) of 5' (left hand panels) and 3' (right hand panels) tRNA fragments found in wild-type (wt) versus NSun2 knockout (-/-) samples. (C) Average frequency of 5' (grey box) and 3'/CCA-tagged tRNA fragments using all expressed tRNAs with (orange line) and without (blue line) a cytosine at positions 48 and 49 in NSun2-expressing mouse (upper panel) and human (lower panel) samples. (D) Isotype-specific enrichment of 5' tRNA fragments (grey box) in NSun2 -/- (orange line) versus respective control (blue line) in mouse (upper panel) and human (lower panel) samples. (E) LC-MS analysis of total $m^5C$ and other common ribonucleoside tRNA modifications. The graph shows the fold change of tRNA modifications in NSun2 -/- relative to wild-type mouse skin. The tRNA modifications are normalized to guanosine. Error bars: SD (n=3). (F) Frequency of RNA sequence errors at each nucleotide in all tRNAs sequence of all tRNAs isolated from in wild-type (blue bars, positive axis) and NSun2 -/- (orange bars, negative axis) skin. Pol III ChIP-seq data from from the respective samples (back dotted line, negative axis) served as genome reference sequence. Potential post-transcriptional tRNA modifications are indicated at the top.

# Supplementary Figure 4

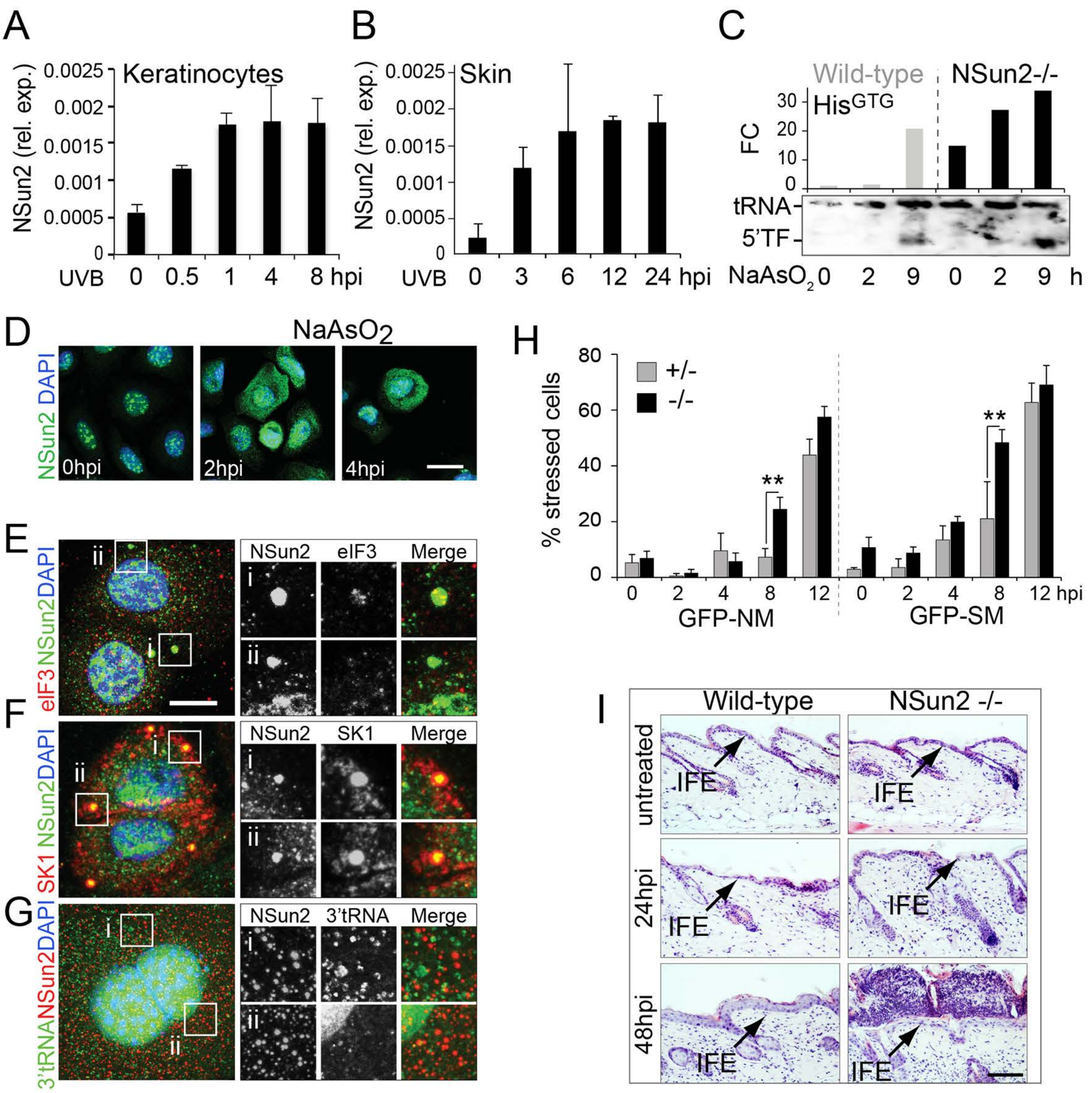


**Figure S4.** NSun2 in the oxidative stress response. (A,B) qPCR of NSun2 mRNA from cultured wild-type mouse keratinocytes (A) or back skin (B) after exposure to UVB. Error bars: SD (n=3). (C) Northern Blot (lower panel) and fold change (upper panel) of 5' tRNA fragments (5'TF) versus full-length tRNA (tRNA) of NSun2 target $His^{GTG}$ tRNA in wild-type and NSun2 -/- mouse keratinocytes treated with $NaAsO_2$ for indicated hours (h). (D) Immunofluorescence of NSun2 (green) in mouse primary keratinocytes after exposure to oxidative stress ($NaAsO_2$) for the indicated hours. (E-G) Co-localization of NSun2 with markers of SGs (eIF3η) (E), PBs (SK1) (F) and 3'tRNA halves in cytoplasmic granules (G) in wild-type mouse primary keratinocytes 4 hours after UV-exposure. (i, ii) Higher magnification of the inserts in the left panels. (H) Percentage of human fibroblasts showing proteome stress in response to $NaAsO_2$ when NSun2 is expressed (+/-) or deleted (-/-). NM: no mutation; SM: single mutation constructs of the GFP-tagged Fluc constructs. Hpi: hours post induction. Error bars: SD (n=3); **$p<0.05$. (I) Haematoxylin and Eosin staining of wild-type and NSun2 -/- mouse back skin untreated (top panels), 24 hours (middle panels) and 48 hours (lower panels) with UVB radiation. Arrows point to IFE: Interfollicular epidermis. Nuclei are counter-stained with DAPI (D-G) and Haematoxylin (I). Scale bar: 25 μm (D-G), 100 μm (I).

# Supplementary Figure 5

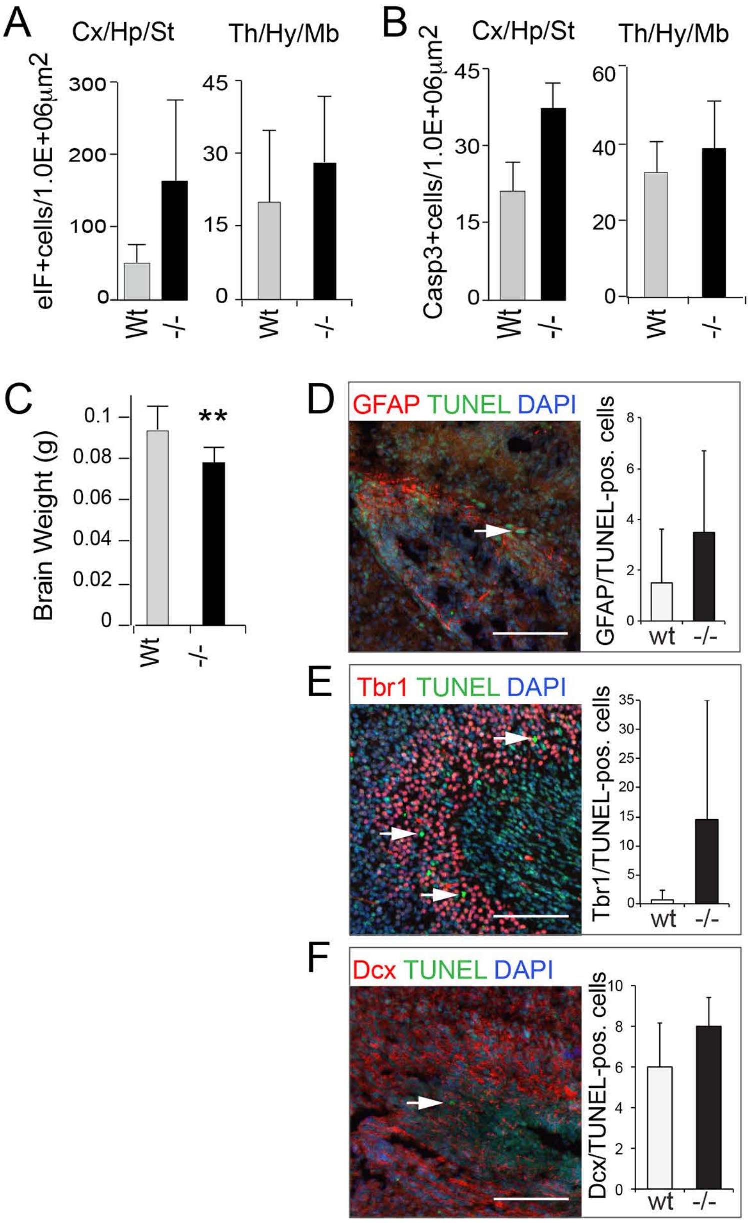


**Figure S5.** NSun2 deletion increases cellular stress and cell death in the brain. (A,B) Automated counting of number of stressed cells (eIF4AI+ cells) (A) or apoptotic cells (Cleaved-Caspase3+ cells) (B) per million $\mu m^2$ in the indicated brain areas. Cx, cortex; Hp, hippocampus; St, striatum; Th, thalamus; Hy, hypothalamus; Mb, midbrain. (C) Weight of wt and NSun2 -/- brains at E18.5. Error bars: SD ($n \geq 3$). ** $p < 0.005$. (D-F) Double staining for markers of apoptosis (TUNEL; green) and glia (GFAP, red) (D) and immature neurons (Tbr1, red) (E) and (Dcx, red) (F) in brain sagittal sections of E18.5 embryos (left panels). Nuclei are stained with DAPI. Arrows indicate TUNEL-positive cells. Quantification of double positive cells in the cortex, hippocampus and striatum are on the right. Scale bar: 100 µm (D-F).

# Supplementary Figure 6

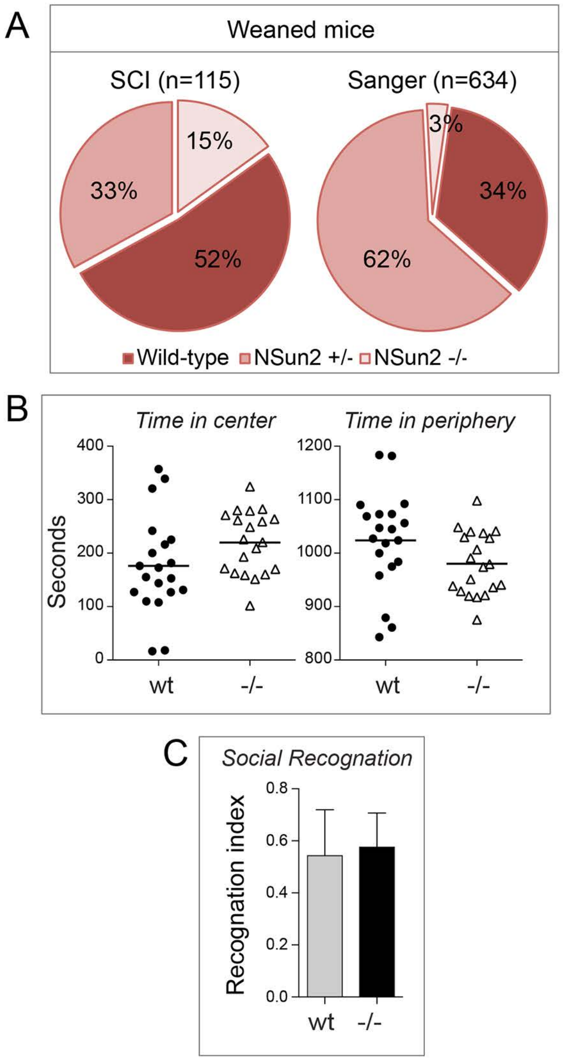


**Figure S6.** Reduced viability of NSun2 -/- adult mice and behavioural analysis. (A) Genotype percentages of weaned mice from Nsun2$^{tm1a(EUCOMM)Wtsi}$ strain, bred for this study (SCI) or at the Wellcome Trust Sanger Institute (Sanger). (B) Time spent in the center (left panel) and in the periphery (right panel) in the open field test. Wt: n=20; (-/-): n=20. (C) Social recognition index using Wt and -/- mice (wt: n=16; -/-: n=29). Error bar: SD.

# Supplementary Figure 7

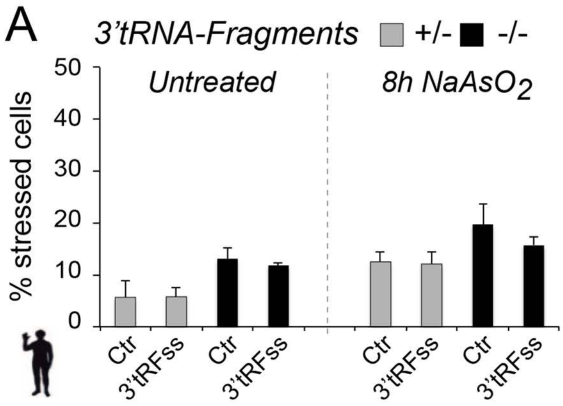


**Figure S7.** 3' tRNA fragments do not induce stress responses. (A) Percentage of NSun2 +/- (grey) and NSun2 -/- (black) fibroblasts with aggregated GFP in response to synthetic sense 3' tRNA fragments (3'tRFss) when untreated (left hand panel) or treated with $NaAsO_2$ (right hand panel). tRF: tRNA fragment; as: anti-sense; ss: synthetic sense; Ctr: mock treated (scrambled RNA). Error bars: SD (n=3).

# Supplementary Figure 8

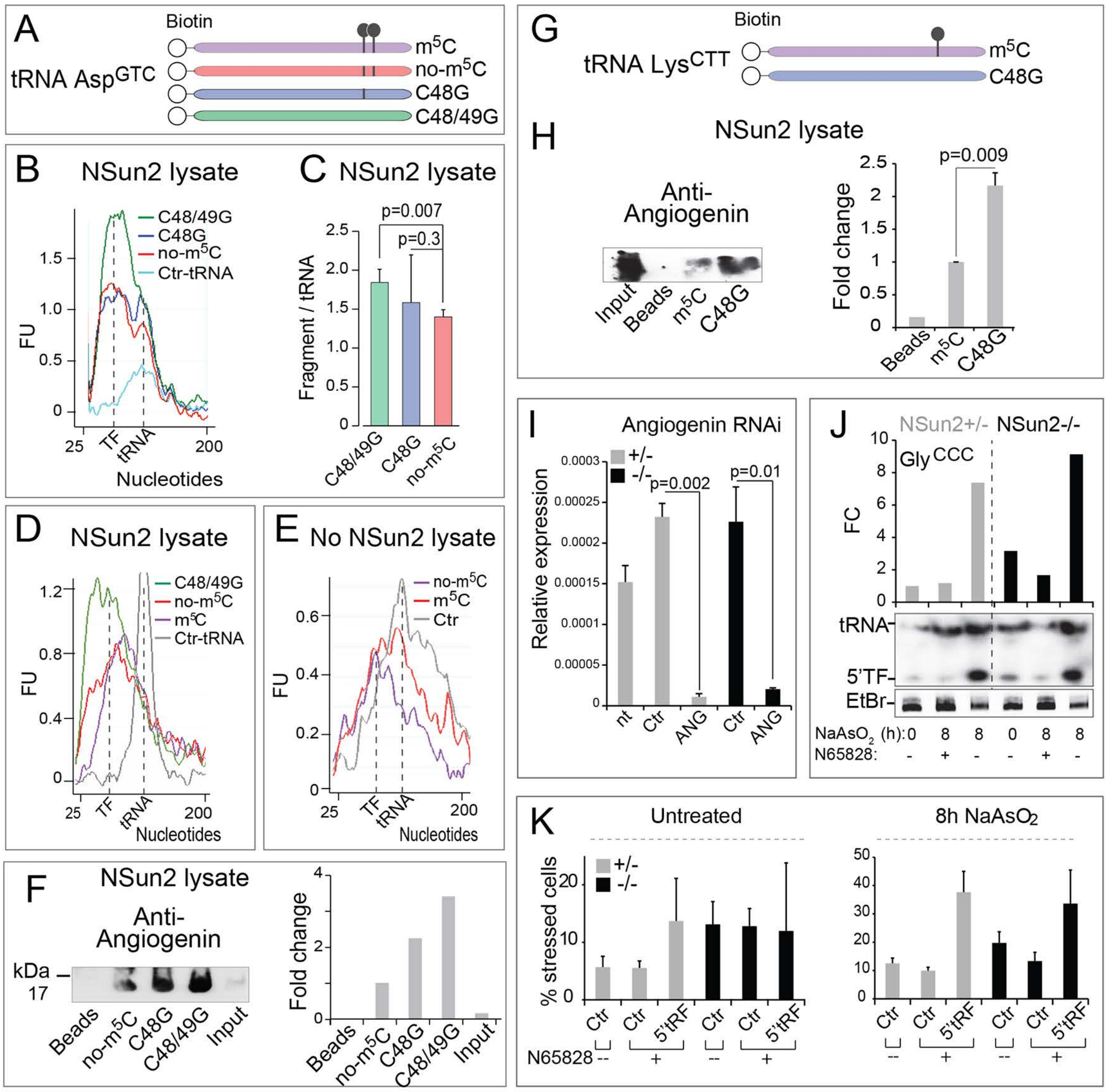

**Figure S8.** NSun2-methylation protects tRNAs from stress-induced cleavage by angiogenin. (A) Scheme of *in vitro* synthesized 5'-biotinylated tRNA $Asp^{GTC}$ constructs with (purple) or without $m^5C$ (red) at position 48 and 49 or substituted cytosines at C48 (C48G; blue) and both C48 and 49 (C48/49G; green). (B-E) Bioanalyzer profiles (B,D,E) and quantification (C) of full length versus cleaved tRNA constructs incubated with NSun2 +/- (B,C,D) or NSun2 -/- (E) cell extracts. Dotted lines indicate full-length and cleaved tRNA (TF) (B,D,E). Ctr-tRNA: biotinylated $m^5C$-$Asp^{GTC}$ not incubated with cell extracts served as control (B-E). FU: arbitrary fluorescence units (B,D,E). (F) Western blot (left hand panel) and quantification (right hand panel) of angiogenin bound to the indicated tRNA $Asp^{GTC}$ construts after incubation with NSun2 +/- cells. (G,H) Scheme (G) and western blot (left hand panel) with quantification (right hand panel) (H) of *in vitro* synthesized 5'-biotinylated tRNA $Lys^{CTT}$ constructs with $m^5C$ (purple) or mutated cytosine C48G (blue) bound to angiogenin in NSun2 +/- cell lysates. tRNAs bound to beads were used as loading control (F,H). Error bars: SD (n=3) (C,H). (I) Relative expression of human angiogenin mRNA 24 hours after angiogenin RNAi transfection in NSun2 +/- and NSun2 -/- fibroblasts. Nt: non-transfected. Ctr: scrambled RNAi control. Error bars: SD (n = 3). (J) Northern blot (bottom panel) and quantification (upper panel) of full-length tRNA and fragments (5'TF) of tRNA $Gly^{CCC}$ relative to total tRNAs (EtBr) in NSun2 +/- and -/- fibroblast after treatment with $NaAsO_2$ for the times (in hours) indicated. Cells were either untreated (-) or treated (+) with the angiogenin inhibitor N65828. (K) Percentage of NSun2 +/- (grey) and NSun2 -/- (black) fibroblasts with aggregated GFP after transfection of 5' tRNA halves in absence (--) or presence (+) of N65828 untreated (left panel) or treated with $NaAsO_2$ for 8 hours (right panel). Error bars: SD (n = 3).

# Supplementary Figure 9

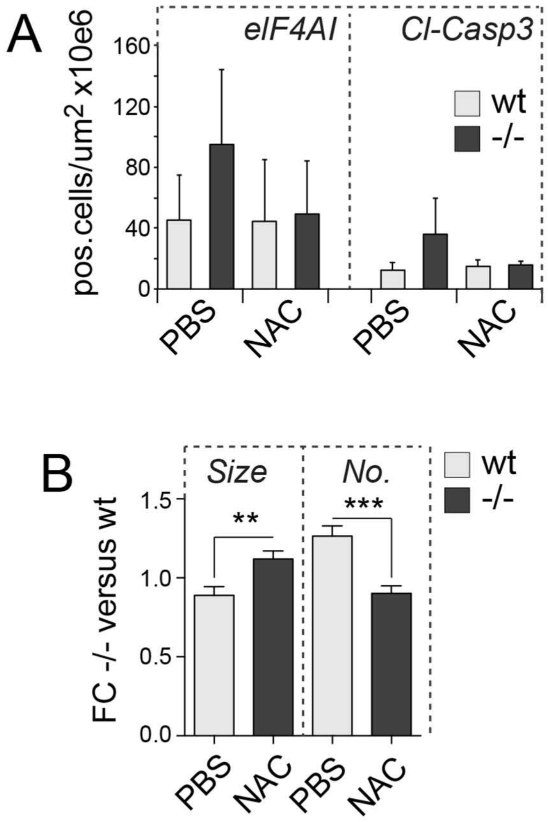


**Figure S9.** N-Acetyl-Cysteine rescues the deleterious effects of loss of NSun2 in mouse embryonic brain *in vivo*. (A) Number of stressed cells (eIF4AI+ cells) (left hand panel) or apoptotic cells (Cleaved Caspase+ cells) (right hand panel) per million $\mu m^2$ in the frontal brain lobe of wild-type (wt) and NSun2 -/- (-/-) embryos after treatment. (B) Fold change of cell size (left hand panel) and number of cell counts per area (right hand panel) of cortical neurons in NSun2 -/- versus wild-type E18.5 embryos after treatment. Error bars: SD ($n \geq 3$) (A) and SEM ($n \geq 3$ mice. 1 mouse: all cells in 7 optical fields); ** $p < 0.01$; *** $p < 0.001$ (B).
