# Supplementary figures and images for: Aberrant methylation of tRNAs links cellular stress to neuro-developmental disorders
Source: EMBO J. 2014 Jul 25;33(18):2020–39. doi: 10.15252/embj.201489282 (PMC4195770; doi:10.15252/embj.201489282)

Figure 6C\_Source data

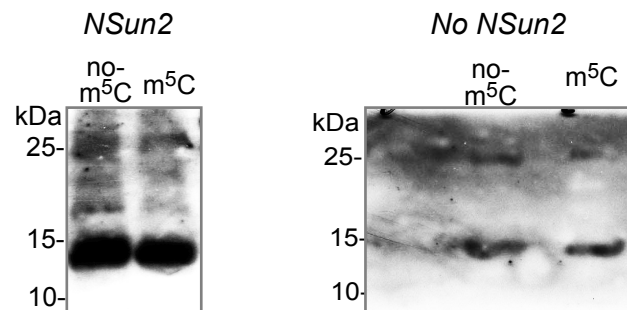

Supplement: Supplementary file 10 [file embj0033-2020-sd10.zip › EMBOJ-2014-89282-source_data_Fig6_blots.pdf]
