# Supplementary material for: Aberrant methylation of tRNAs links cellular stress to neuro-developmental disorders
Source: EMBO J. 2014 Jul 25;33(18):2020–39. doi: 10.15252/embj.201489282 (PMC4195770; doi:10.15252/embj.201489282)
Supplement: Supplementary file 6 [file embj0033-2020-sd6.pdf]

THE
EMBO
JOURNAL

Manuscript EMBO-2014-89282

Aberrant methylation of tRNAs links cellular stress to neuro-developmental disorders

Sandra Blanco, Sabine Dietmann, Joana V. Flores, Shobbir Hussain, Claudia Kutter, Peter Humphreys, Margus Lukk, Patrick Lombard, Lucas Treps, Martyna Popis, Stefanie Kellner, Sabine M. Hölter, Lillian Garrett, Wolfgang Wurst, Lore Becker, Thomas Klopstock, Helmut Fuchs, Valerie Gailus-Durner, Martin Hrabě de Angelis, Ragnhildur T. Káradóttir, Mark Helm, Jernej Ule, Joseph G. Gleeson, Duncan T. Odom, Michaela Frye

Corresponding author: Michaela Frye, University of Cambridge

Review timeline:

Submission date:	16 June 2014
Accepted:	23 June 2014

Transaction Report:

(Note: With the exception of the correction of typographical or spelling errors that could be a source of ambiguity, letters and reports are not edited. The original formatting of letters and referee reports may not be reflected in this compilation.)

Editor: Thomas Schwarz-Romond

Transfer Note 23 June 2014

PLEASE NOTE that this manuscript was transferred from a different journal and the arbitrating referee assessing suitability for The EMBO Journal had access to both the original anonymous comments as well as the point by point response provided by the authors.

Editorial Staff
The EMBO Journal

1st Editorial Decision 23 June 2014

I am pleased to inform you that your manuscript has been accepted for publication in the EMBO Journal.

- However, before we can transfer your manuscript data to our publisher we need an amended article file (in Word format) including the sections "Materials and Methods" (is currently a supplementary file), "Conflict of interest" and "Author contributions".

- The EMBO Journal further encourages the publication of source data, particularly for electrophoretic gels/blots and excel tables underlying the graphs, with the aim to make primary data more accessible and transparent to the reader. We would be grateful for one PDF-file per figure with such information, respective transmission of relevant excel sheets used to plot the graphs (at least for those supporting the main findings of the paper) . These will be linked online as "Source Data" files and available for the interested community.

- Lastly, we are also in the position to highlight the major outcome of the paper in a written, 2-4 bullet point synopsis AND a summarising scheme for better graphical perception (like a 'graphical abstract').

I would be delighted if you were to provide such items to your earliest convenience as to ensure efficient production/publication of your study.

Please send these files to us via email to t.schwarz-romond@embojournal.org and do not hesitate to get in touch in case of further questions!

Thank you very much in advance.

Referee Comments:

Referee #1:

RNA modifications represent a timely and emerging topic in the field of (epigenetic) gene regulation. In this study, Blanco et al. provide a detailed molecular and phenotypic characterization of human and mouse cells and animals that are deficient for the cytosine-RNA methyltransferase NSUN2. Mutations in NSUN2 have previously been linked to neurodevelopmental disorders and intellectual disability, which has raised considerable interest in the underlying molecular mechanisms. The manuscript contains 3 major lines of analysis, each with significant novel findings: (1) A comprehensive analysis of transcriptome-wide methylation datasets reveals that NSUN2-mediated tRNA methylation at C48/49/50 is a common and conserved modification. (2) Results from various molecular approaches suggest that the lack of tRNA methylation in NSUN2-deficient cells causes the accumulation of 5'-tRNA fragments through increased angiogenin cleavage. In agreement with the known role of 5'-tRNA fragments in stress signalling, NSUN2-deficient cells showed globally reduced rates of protein translation. (3) A thourough phenotypic analysis of NSUN2-deficient mice revealed various neurological and neurodevelopmental defects that could be linked to reduced tRNA methylation and the increased accumulation of 5'-tRNA fragments. Interestingly, several aberrant phenotypes of NSUN2-defiecient mice and cells could be rescued by the inhibition of angiogenin, a ribonuclease that preferentially cleaves unmethylated tRNAs.

This is a polished manuscript with an interesting story that contains a wealth of novel data. The quality of the data appears very high, even if the interfaces between the 3 main lines of experimentation will require additional work in future studies. Cell has recently published two manuscripts that linked neurodevelopmental disorders to tRNA biogenesis (PMID: 24766810; PMID: 24766809). However, the manuscript by Blanco et al. clearly is conceptually distinct from these publications and also adds tRNA methylation as an exciting additional regulatory mechanism.

I would also like to mention that I have been provided with the existing referee comments from peer-review at an alternative journal. I have thoroughly considered the responses of the authors and I feel that the comments raised by the reviewers were adequately addressed. As such, I would consider the manuscript by Blanco et al. as immediately suitable for publication in EMBO J.
